# Heat Capacities of *N-*Acetyl Amides of Glycine, L-Alanine, L-Valine, L-Isoleucine, and L-Leucine

**DOI:** 10.3390/molecules28145440

**Published:** 2023-07-16

**Authors:** Vojtěch Štejfa, Václav Pokorný, Eliška Lieberzeitová, Jakub Havlín, Michal Fulem, Květoslav Růžička

**Affiliations:** 1 Department of Physical Chemistry, University of Chemistry and Technology, Prague, Technická 5, CZ-166 28 Prague, Czech Republic; stejfav@vscht.cz (V.Š.); pokorny@imc.cas.cz (V.P.); e.lieberzeit@gmail.com (E.L.); fulemm@vscht.cz (M.F.); 2 Institute of Macromolecular Chemistry, Czech Academy of Sciences, Heyrovského nám. 2, CZ-162 06 Prague, Czech Republic; 3 Central Laboratories, University of Chemistry and Technology, Prague, Technická 5, CZ-166 28 Prague, Czech Republic; havlinj@vscht.cz

**Keywords:** *N-*acetyl glycine amide, *N-*acetyl-L-alanine amide, *N-*acetyl-L-valine amide, *N-*acetyl-L-isoleucine amide, *N-*acetyl-L-leucine amide, crystalline phase, heat capacity

## Abstract

As a follow-up to our effort to establish reliable thermodynamic data for amino acids, the heat capacity and phase behavior are reported for *N*-acetyl glycine amide (CAS RN: 2620-63-5), *N*-acetyl-L-alanine amide (CAS RN: 15962-47-7), *N*-acetyl-L-valine amide (CAS RN: 37933-88-3), *N*-acetyl-L-isoleucine amide (CAS RN: 56711-06-9), and *N*-acetyl-L-leucine amide (CAS RN: 28529-34-2). Prior to heat capacity measurement, thermogravimetric analysis and X-ray powder diffraction were performed to determine decomposition temperatures and initial crystal structures, respectively. The crystal heat capacities of the five *N*-acetyl amino acid amides were measured by Tian–Calvet calorimetry in the temperature interval (266–350 K), by power compensation DSC in the temperature interval (216–471 K), and by relaxation (heat-pulse) calorimetry in the temperature interval (2–268 K). As a result, reference heat capacities and thermodynamic functions for the crystalline phase from 0 K up to 470 K were developed.

## 1. Introduction

This work is an extension of our project, the goal of which is to establish reliable thermodynamic data along the saturation curve for a group of proteinogenic amino acids [1,2,3,4,5,6] and their derivatives.

The zwitterionic structure is disrupted in amino acid derivatives, resulting in significant changes in their chemical and physicochemical properties. Amino acid derivatives are typical representatives of small uncharged proteins, which can be used as model molecules for studying, for example, solute-solvent interactions in aqueous media [7] or interactions occurring in globular proteins [8].

In this work, the phase (polymorphic) behavior and heat capacity of five *N*-acetyl amide derivatives of aliphatic amino acids (glycine, L-alanine, L-valine, L-isoleucine, and L-leucine) were studied, which represent essential properties indispensable for studying the physical stability of the respective crystalline phases, evaluating the temperature dependence of thermodynamic properties such as the sublimation enthalpy, and evaluating the solvation or hydration enthalpies. First, the initial crystal structures of the title compounds were verified by X-ray powder diffraction (XRPD), and their melting or decomposition temperatures were studied by thermogravimetry. Subsequently, the phase behavior was studied using heat flux differential scanning calorimetry (DSC) to detect pos-sible phase transitions. Heat capacities in the temperature range (266 to 350 K) were determined with Tian-Calvet calorimetry and extended down to 2 K using relaxation calorimetry and up to 471 K using power compensation DSC. Heat capacities measured using a less accurate heat flux DSC by Abate et al. [7] were found in the literature, but they were not considered in the final correlation of heat capacities due to their higher uncertainty. Based on the obtained heat capacity data and phase behavior, fundamental thermodynamic functions (entropy, enthalpy, and Gibbs energy) were calculated for the crystalline phases from 0 K to 470 K. 

## 2. Results and Discussion

### 2.1. Thermogravimetric Analysis (TGA)

**Figure 1:**
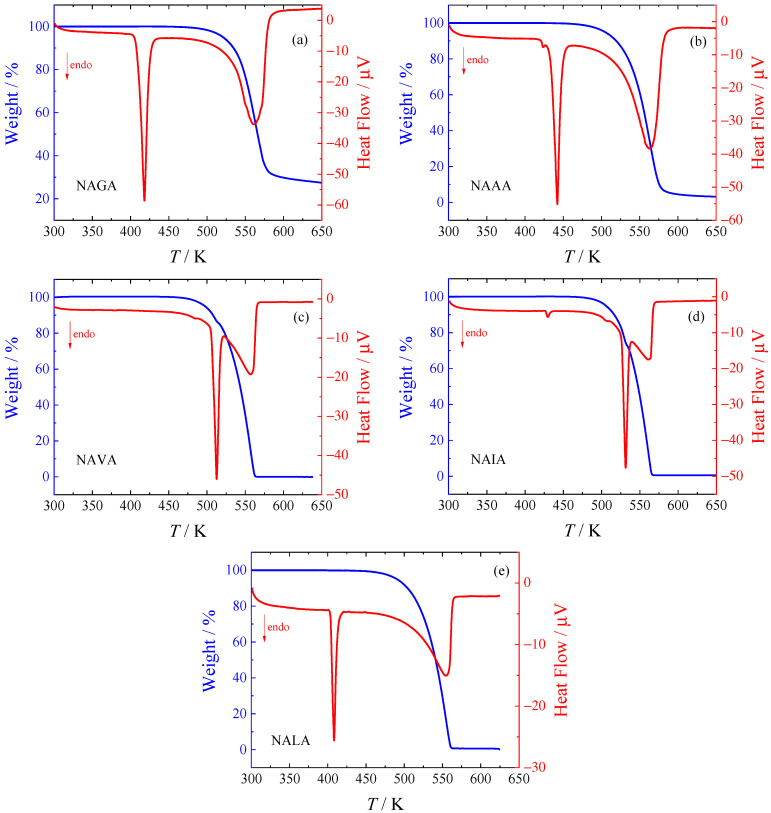
TGA analysis of studied *N*-acetyl amino acid amides. (**a**) *N-*acetyl-glycine amide; (**b**) *N-*acetyl-L-alanine amide; (**c**) *N-*acetyl-L-valine amide; (**d**) *N-*acetyl-L-isoleucine amide; (**e**) *N-*acetyl-L-leucine amide.

All samples were studied by TGA, and the results are shown in Figure 1. The decomposition temperatures evaluated as the onset temperatures of the decomposition peaks on the TGA heat flow curve are summarized in Table 1. The fusion temperatures were also determined by TGA, and they are compared with the DSC results in the next section.

All studied *N*-acetyl amino acid amides undergo a simple one-step decomposition; however, for NAVA and NAIA, the decomposition occurs simultaneously with melting. Peaks of melting and decomposition can be clearly distinguished in Figure 1; the melting peak is sharp and symmetrical, while the decomposition peak is wide and tilted to the right (towards higher temperatures). Note that the final mass of NAGA is ca. 25 percent of the original mass (see Figure 1), which is substantially higher than for the remaining compounds. The second decomposition step at temperatures above 623 K cannot thus be excluded.

### 2.2. Phase Behavior

**Figure 2:**
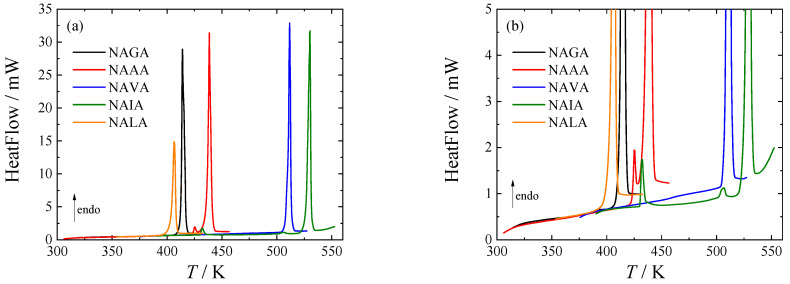
DSC curves of studied *N*-acetyl amino acid amides. (**a**) Full scale; (**b**) baseline detail. For an explanation of the abbreviations, see Table 1.

All title compounds are crystalline at 298.15 K, and their crystal structures identified by XRPD are provided in Table 2. Subsequently, the phase behavior was investigated in the temperature range from 183 K to the thermal decomposition temperature using heat-flux DSC to confirm/exclude the presence of phase transitions. Records from the first DSC runs are shown in Figure 2. Temperatures and enthalpies of fusion are compared to TGA results and literature values in Table 1. The crystal-crystal phase transitions observed in this work are listed and compared with literature values in Table 3.

For NAVA and NAIA, the onset of decomposition occurs prior to their melting, and the ability of the partially decomposed sample to recrystallize is very poor. Fusion temperatures and enthalpies could be collected from runs with a fresh sample, and their uncertainty was properly increased. However, even for the remaining compounds that decompose far above their melting temperatures according to TGA, slow decomposition was observed when determining fusion temperatures repeatedly. Even here, fusion temperatures and enthalpies were again taken from first runs, where possible. For polymorphs obtained by recrystallization from melt, the uncertainties were increased to account for the lower purity of the sample.

Taking into account the uncertainty of the melting properties due to decomposition, agreement between DSC, TGA, and literature fusion temperatures and enthalpies is generally good for NAGA (phase α), NAAA, and NAVA. Another phase of NAGA (phase β) that was found to preferably crystallize from melt (see Figure S2 in the Supplementary Materials) was probably not observed before. A positive correlation between the fusion enthalpies and temperatures for a single compound from various sources can be noticed, which is probably a result of using materials with different purities. Repeated melting experiments performed in this work confirmed that the decrease in fusion enthalpy and temperature due to decomposition exhibit a linear correlation in a certain range of impurities.

A small endothermal peak before the melting of NAAA was observed at 424 K (see Figure S3 in the Supplementary Materials). The reversibility of this enantiotropic crII-crI phase transition is puzzling. At cooling, the reverse transition occurs at around 408 K, but during repeated cycles, areas of both (heating and cooling) peaks decrease more rapidly than would be expected due to the slow decomposition at the given temperatures. After the first melting, the peak was never observed again, although it seemed that it was always the same phase (crI) that melted.

For NAVA, a small endothermic peak was observed at 477.6 ± 2.0 K, but only in one of the two runs with a fresh sample (Figure S4 in the Supplementary Materials). The initial crystalline structure of the sample was not identified with CSD ref code JEXNAB [11], but rather with a material containing this structure together with another unresolved polymorph. Therefore, it is possible that the commercial material is a mixture of different crystals, and the occurrence of the transition depends on the polymorph ratio in the used sample.

For NAIA, two endothermic phase transitions at 429.3 ± 1.0 K and 502.0 ± 1.0 K were observed (Figure S5 in the Supplementary Materials). The first one (crIII-crII) was found to be fairly reversible. The reversibility of the latter one (crII-crI) was not tested since it occurred in the region where the decomposition of the sample proceeds. Neither of these transitions was reported in the previous study [10]. At the same time, the fusion enthalpy and temperature reported in [10] differ considerably (+3.1 K and −9%, respectively) from our values. Since the deviations in the fusion enthalpy and temperature do not follow the same direction, the observed difference is likely not due to sample purity, but the previously reported values may belong to another (fourth) polymorph.

The phase transition reported for NALA at 382 K [7,10] was not observed in this work using DSC or TGA. Instead, melting of two different polymorphs that can occur separately or concomitantly was observed (see Figure S6 in the Supplementary Materials). Based on the fusion temperatures and enthalpies of these two polymorphs, they should be related monotropically, and the reported phase transition [7,10] may not correspond to the transformation of one of them to the other. Although the XRPD diffractogram of the sample studied did not fit the known structure of JAHZUN10 [12] and might correspond to a mixture of polymorphs or to an incompletely or imperfectly crystallized sample, the melting peak observed in the first run was smooth and sharp, which is a sign of a crystalline phase with high purity. The fusion enthalpies of NALA reported previously [7,9,10] are lower than our results, which might be explained by our observation that melting of the concomitant mixture exhibited a lower enthalpy than both of the pure phases.

### 2.3. Heat Capacities

Experimental heat capacities obtained in this work with SETARAM μDSC IIIa, PerkinElmer DSC 8500, and Quantum Design PPMS are listed in Tables S1–S10 in the Supplementary Materials, including correction scaling factors applied for PerkinElmer DSC 8500 and Quantum Design PPMS results. For NAGA, NAAA, and NALA, liquid phase heat capacity data of reasonable quality were obtained despite the slow decomposition in the liquid phase. For NAIA, heat capacities of both crIII (below 429 K) and crII (above 429 K) were obtained. Available literature data on crystal heat capacities are summarized in Table 4. To describe the temperature dependence of selected heat capacity data from Table 4, the following equation proposed by Archer [14] was used, the parameters of which are given in Table 5:

(1) $$C_{pm}^{o}/C_{pm}^{\mathrm{ref}}={\left(\frac{T}{T^{\mathrm{ref}}f(T)+bT}\right)}^{3}$$

where $T^{\mathrm{ref}}$ = 1 K, $C_{pm}^{\mathrm{ref}}$ = 1 J∙K^−1^∙mol^−1^, and

(2) $$f(T)=a_{i}{\left(T-T_{i}\right)}^{3}+b_{i}{\left(T-T_{i}\right)}^{2}+c_{i}\left(T-T_{i}\right)+d_{i}$$

Only a single parameter, *d_i_*, per each temperature interval is to be optimized, while the values of the other three are imposed by a constraint on the continuity and smoothness of the resulting temperature dependence. Parameter *b* can be estimated from the slope of f(T) at temperatures greater than 70 K prior to the optimization procedure [14]; the universal value *b* = 0.13 K^−1^ was used in this work.

**Figure 3:**
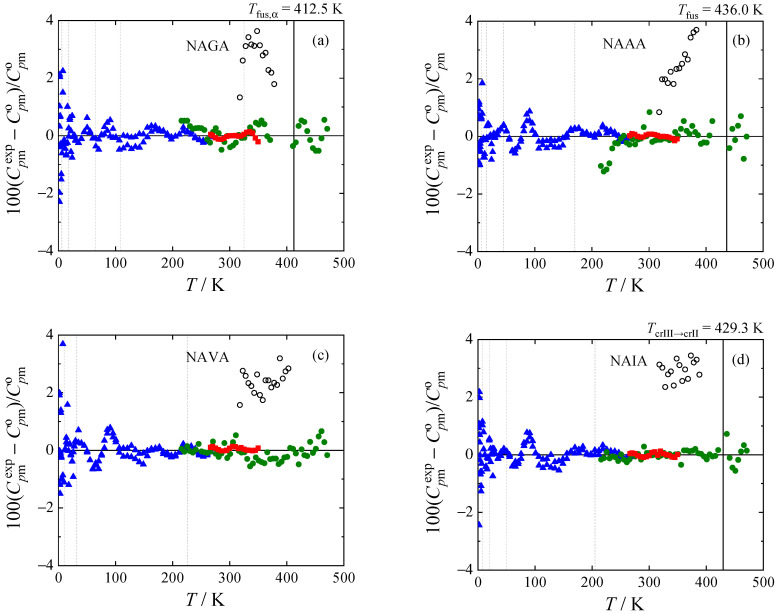

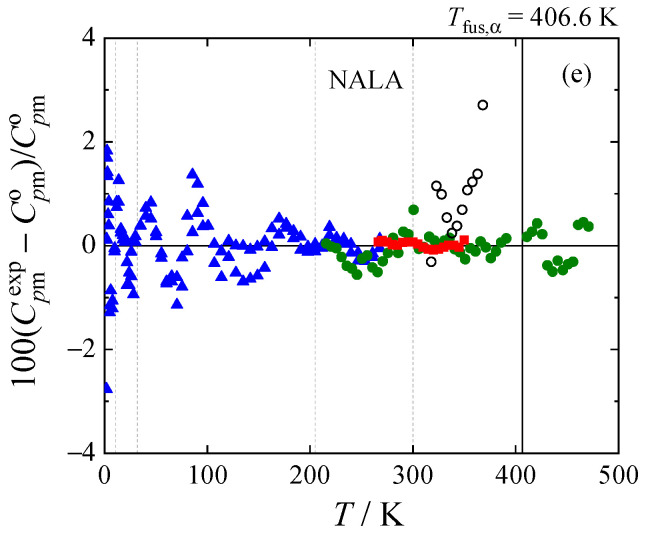
Relative deviations $100\left(C_{pm}^{\exp}-C_{pm}^{o}\right)/C_{pm}^{o}$ of individual experimental heat capacities $C_{pm}^{\exp}$ from values $C_{pm}^{o}$ calculated by means of Equations (1) and (2) with parameters from Table 5. (**a**) *N-*acetyl glycine amide; (**b**) *N-*acetyl-L-alanine amide; (**c**) *N-*acetyl-L-valine amide; (**d**) *N-*acetyl-L-isoleucine amide; and (**e**) *N-*acetyl-L-leucine amide. Blue, This work (relaxation calorimetry); red, this work (Tian-Calvet calorimetry); green, this work (power compensation DSC); hollow, Abate et al. [7] (heat flux DSC). Vertical lines mark knot temperatures *T_i_* with thick lines marking phase transitions. Data points represented by filled symbols were used to obtain the parameters of Equations (1) and (2).

The experimental heat capacities for all *N*-acetyl amino acid amides studied are compared with the smoothed values obtained using Equations (1) and (2) in Figure 3. The deviations of the selected experimental data (marked bold in Table 4) from the smoothed values do not exceed 2% (with the exception of the lowest temperatures, where all experimental methods naturally have higher relative uncertainty).

The literature data by Abate et al. [7] obtained by heat-flux DSC (Mettler DSC 20) are about 3% higher compared to our data, with the exception of NALA, where the agreement is mostly within 1%. The comparison is complicated by the fact that all the compounds seem to occur in several crystalline forms, and in the previous work, the respective crystalline forms were not reported. The same authors resolved the crystalline structures of all five *N*-acetyl amino acid amides [11,12,13], but the studied single crystals were prepared specifically for the X-ray experiments and do not need to correspond to the polymorphs studied calorimetrically. Generally, the uncertainty of heat capacity determination by heat-flux DSC is several percent at best, and thus the agreement should be considered reasonable.

**Figure 4:**
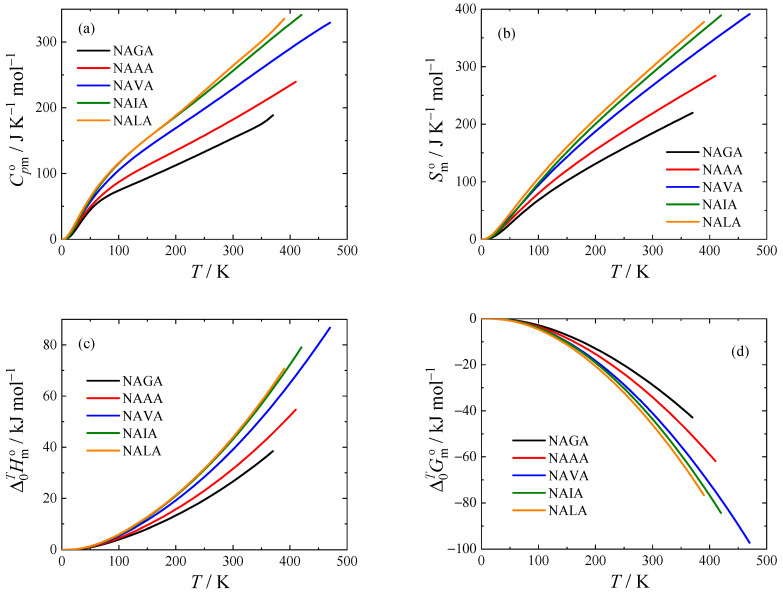
Standard molar thermodynamic functions of crystalline *N*-acetyl amides at *p* = 0.1 MPa. (**a**) isobaric heat capacity; (**b**) entropy; (**c**) enthalpy; and (**d**) Gibbs energy. For an explanation of the abbreviations, see Table 1.

The thermodynamic functions of all studied compounds at *T* = 298.15 K obtained using Equations (1) and (2) are tabulated in Table 6; for thermodynamic functions in a wide temperature range (from 0 K to up to 470 K, depending on the thermodynamic stability of the crystalline phase), see Figure 4 and Tables S11–S15 in the Supplementary Materials.

**Figure 5:**
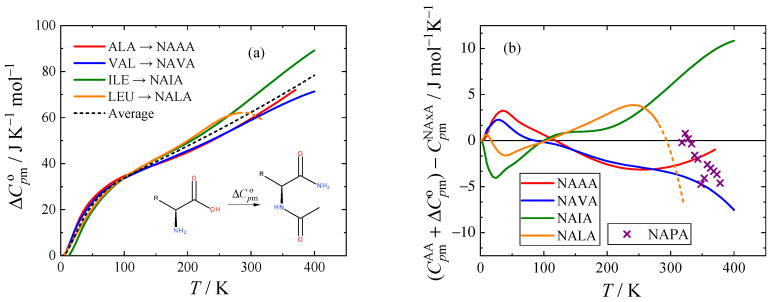
(**a**) Difference of isobaric heat capacity Δ$C_{pm}^{o}$ between aliphatic amino acids and their *N-*acetyl amides. (**b**) Absolute deviations of isobaric heat capacities of *N-*acetyl amides estimated using the experimental heat capacity of the respective amino acid ($C_{pm}^{\mathrm{AA}}$) [1] and the average Δ$C_{pm}^{o}$ from the experimental heat capacity of the *N-*acetyl amide $C_{pm}^{\mathrm{NAxA}}$. Average Δ$C_{pm}^{o}$ is represented by the correlation equation (Equations (1) and (2) with parameters from Table 7). The dashed line highlights the region where the data for L-leucine were excluded because of a lambda phase transition. For the *N-*acetyl amide abbreviations, see Table 1; NAPA is *N*-acetyl-L-proline amide.

The differences in molar isobaric heat capacity Δ$C_{pm}^{o}$ between aliphatic amino acids presented by Pokorný et al. [1] (L-alanine, L-valine, L-isoleucine, and L-leucine) and their *N*-acetyl amides were calculated, and they are shown in Figure 5a. Although the heat capacities of glycine are also available [15], glycine was not included because of the existence of several polymorphs and its rather outlying character compared to the other proteinogenic amino acids. An average value of the difference was then fitted to the Archer equation (Equations (1) and (2)), the parameters of which are presented in Table 7. Data influenced by the lambda transition of L-leucine [1] were also not considered in the average. For NALA and NAVA, the Δ$C_{pm}^{o}$ trend above 280 and 370 K, respectively, was approximated by a linear function based on a 100 K interval to avoid discontinuity of the curve and a bias towards compounds measured up to higher temperatures.

Deviations of isobaric heat capacities of *N-*acetyl amides calculated using the correlation of the average difference between amino acids and their *N-*acetyl amides and the experimental heat capacities of the respective amino acids are shown in Figure 5b. The heat capacity estimated in this way falls within 10 J mol^−1^ K^−1^ (and within 5% of the experimental values above 50 K) for all tested compounds. The parameters in Table 7 could therefore serve as a reasonable estimation for the *N-*acetyl amides of the remaining amino acids above 50 K.

The predictive ability of this correlation was tested on *N*-acetyl-L-proline amide (NAPA) and L-proline. The heat capacities used for these compounds were taken from Abate et al. [7] and our previous publication [2], respectively. As shown in Figure 5b, the predicted heat capacities of NAPA fall within 5 J K^−1^ mol^−1^ (3% in relative scale) of the estimation, which is well in agreement with the expected accuracy of the estimation and also of the experimental heat capacity data [7].

## 3. Materials and Methods

### 3.1. Samples Description

The title *N*-acetyl amino acid amides were of commercial origin and were used as received. The sample characteristics are reported in Table 8.

### 3.2. Thermogravimetry

While amino acids are known to decompose at temperatures prior to melting [16], temperatures and enthalpies of fusion were previously reported for all *N*-acetyl amides studied in this work [7]. In order to safely reinvestigate the melting properties and heat capacities at elevated temperatures, thermogravimetric analysis (TGA) was performed at first. The thermogravimetric analyzer SETARAM Setsys Evolution was used. All samples were placed in an open platinum 100 μL crucible employing the temperature range (298 to 573) K with a temperature gradient of 5 K min^−1^ under an inert Ar atmosphere.

### 3.3. Phase Behavior Study

XRPD was used to characterize the initial crystal structures of the *N*-acetyl amides using a *θ*–*θ* powder diffractometer X’Pert3 Powder from PANalytical in Bragg-Brentano para-focusing geometry using wavelength CuKα radiation (*λ* = 1.5418 Å, *U* = 40 kV, *I* = 30 mA). The samples were scanned at 298.15 K in the range of 5° to 50° 2*θ* with a step size of 0.039° 2*θ* and 0.7 s for each step. The diffractograms were analyzed with the software HighScore Plus in combination with the yearly updated powder diffraction databases PDF4+ and PDF4/Organics.

The heat flux DSC TA Q1000 was used for the investigation of the phase behavior of the *N*-acetyl amides studied in the temperature range from 183 K to the fusion/decomposition temperature. The combined expanded uncertainties (0.95 level of confidence) of the phase transition temperatures and enthalpies are listed in Table 1 and Table 3.

### 3.4. Heat Capacity Measurements

A Tian–Calvet type calorimeter (SETARAM μDSC IIIa) was used for the measurement of heat capacities in the temperature range from 266 K to 353 K. As the detailed description of the calorimeter and its calibration and operation were reported previously [1], only the most salient information is provided here. The heat capacity measurements were carried out by the continuous heating method [17], using the three-step methodology, i.e., the measurement of the sample is followed by the measurement of the reference material (synthetic sapphire, NIST Standard Reference Material No. 720), and by performing a blank experiment. The saturated molar heat capacities *C*_sat_ obtained in this work are identical to isobaric molar heat capacities $C_{pm}^{o}$ in the temperature range studied, given the very low sublimation pressure of the samples. The combined expanded uncertainty (0.95 level of confidence) of the heat capacity measurements is estimated to be $U_{c}(C_{pm}^{o})=0.01C_{pm}^{o}$.

The PerkinElmer power compensation DSC 8500 equipped with an autosampler was used for the heat capacity determination in the temperature range (216 to 471 K). The heat capacity measurements were carried out by the temperature increment method, repeated three times to eliminate systematic errors. The combined expanded uncertainty (0.95 level of confidence) of the heat capacity measurement is estimated to be $U_{c}(C_{pm}^{o})=0.03C_{pm}^{o}$. Due to its lower accuracy, results from this calorimeter were slightly adjusted to agree with those from the more accurate Tian–Calvet type calorimeter, following common practice [18]. Scaling factors are presented in tables containing experimental heat capacities (see Supporting Materials, Tables S1, S3, S5, S7, and S9), and the maximum correction amounted to 0.037$C_{pm}^{o}$ in case of NAVA. The same procedure was also applied for low-temperature relaxation calorimetry, as described in the next paragraph (see Supporting Materials Tables S2, S4, S6, S8, and S10).

For low-temperature heat capacity measurements, a commercially available apparatus, the Physical Property Measurement System (PPMS) Model 6000 EverCool II (Quantum Design, San Diego, CA, USA), equipped with a heat capacity module (^4^He, *T*_min_ = 1.8 K), was used. The calorimeter uses a thermal-relaxation measurement technique, which is an alternative to time- and labor-intensive adiabatic calorimetry. The specific heat capacity of a sample is determined by measuring the thermal response to a change in heating conditions [19]. Samples were placed in Cu cups (height 3.5 mm, diameter 3 mm) made from 0.025 mm thick copper foil (Alfa Aesar, purity: mass fraction purity 0.99999) by a technique similar to Shi et al. [20]. In contrast to Shi et al. [20], samples were not mixed with Apiezon N; instead, samples enclosed in a Cu cup were pressed to a height of ca. 1 mm using a stainless steel die (Maassen, Reutlingen, Germany) and a press (Trystom, Olomouc, Czech Republic) using a force of 15 kN. The heat capacity of the Cu cup was subtracted from the total heat capacity using data recommended by Arblaster [21]. This technique was checked by measuring compounds with reliable data obtained by adiabatic calorimetry (anthracene [22], L-asparagine [23], and glycine [24]), and the uncertainty of results was found to be comparable to that reported previously [25] for samples encapsulated in Al DSC pans. The combined expanded uncertainty (0.95 level of confidence) of the heat capacity measurements is estimated to be $U_{c}(C_{pm}^{o})=0.10C_{pm}^{o}$ below 10 K, $U_{c}(C_{pm}^{o})=0.03C_{pm}^{o}$ in the temperature range (10 to 40 K), and $U_{c}(C_{pm}^{o})=0.02C_{pm}^{o}$ in the temperature range (40 to 300 K).

## 4. Conclusions

Thermochemical data (namely phase behavior, temperature of melting and decomposition, heat capacity, and other derived thermodynamic functions) of *N*-acetyl amides of aliphatic amino acids were determined in a wide temperature range using TGA, DSC, Tian–Calvet and relaxation calorimetry. The heat capacities are presented in the form of parameters of an inverse spline function valid from 0 K towards the temperature of decomposition.

Unlike amino acids, *N*-acetyl amides melt prior to their complete decomposition. For *N-*acetyl-L-glycine amide, *N-*acetyl-L-alanine amide, and *N-*acetyl-L-leucine amide, fusion properties and heat capacities of the liquid phase could be determined, although slow decomposition was detected during these experiments in the form of a melting temperature decrease with repeated cycles. For *N-*acetyl-L-valine amide and *N-*acetyl-L-isoleucine amide, fusion properties could be determined with somewhat higher uncertainty, but not the liquid heat capacities, since these compounds decompose quite rapidly already at their melting temperature.

The crystalline molar heat capacity of the *N*-acetyl amides is higher compared to the amino acids by a similar amount for all of the studied compounds. A universal contribution function was therefore established, which can be used for estimating the heat capacity of *N*-acetyl amides of other amino acids with reasonable accuracy. For four of the compounds studied in this work (as well as *N-*acetyl-L-proline amide, which was used for validation), this estimation lies within 5% of the experimental values above 50 K.

## Figures and Tables

**Table 1:** Fusion Temperatures (*T*_fus_) and Enthalpies (Δ_fus_*H*) and Decomposition Temperatures (*T*_decomp_) of *N*-Acetyl Amides Studied ^a^.

Reference	*T*_fus_/K	Δ_fus_*H*/kJ/mol	*T*_decomp_/K	Method	Heating Rate, Purge Gas
*N*-acetyl glycine amide (NAGA)					
Abate el al. [7]	412.2 ± 0.2	26.71 ± 0.12		DSC	1 to 2 K min^−1^, nosp. ^b^
Ferro et al. [9]	408.2 ± 0.3	25.6 ± 0.4		DSC	1 K min^−1^, nosp. ^b^
Barone et al. [10]	410.9 ± 0.1	27.0 ± 0.1		DSC	2 K min^−1^, nosp. ^b^
This work (phase α)	412.5 ± 0.7	28.4 ± 0.9		DSC	5 K min^−1^, nitrogen
This work (phase β)	403.1 ± 1.0	22.3 ± 0.9		DSC	5 K min^−1^, nitrogen
This work (phase α)	410		530	TGA HeatFlow	5 K min^−1^, argon
*N*-acetyl-L-alanine amide (NAAA)					
Abate el al. [7]	436.4 ± 0.2	23.59 ± 0.13		DSC	1 to 2 K min^−1^, nosp. ^b^
Ferro et al. [9]	431.0 ± 0.4	21.7 ± 0.2	505	DSC	1 K min^−1^, nosp. ^b^
Barone et al. [10]	435.4 ± 0.1	23.7 ± 0.3		DSC	2 K min^−1^, nosp. ^b^
This work (phase crI)	436.0 ± 0.7	25.3 ± 1.0		DSC	5 K min^−1^, nitrogen
This work (phase crI)	435		519	TGA HeatFlow	5 K min^−1^, argon
*N*-acetyl-L-valine amide (NAVA)					
Abate el al. [7]	509.0 ± 0.3	39.10 ± 0.23		DSC	1 to 2 K min^−1^, nosp. ^b^
Puliti et al. [11]	509.0 ± 0.4	41.3 ± 0.6		DSC	1 K min^−1^, nosp. ^b^
Barone et al. [10]	509.0 ± 0.2	36.9 ± 0.4		DSC	2 K min^−1^, nosp. ^b^
This work	510.1 ± 1.5 ^c^	40.8 ± 2.0		DSC	5 K min^−1^, nitrogen
This work	506		510	TGA HeatFlow	5 K min^−1^, argon
*N*-acetyl-L-isoleucine amide (NAIA)					
Barone et al. [10]	529.6 ± 0.2	41.8 ± 0.1		DSC	2 K min^−1^, nosp. ^b^
This work (phase crI)	526.5 ± 1.5 ^c^	46.2 ± 2.0		DSC	5 K min^−1^, nitrogen
This work (phase crI)	526		512	TGA HeatFlow	5 K min^−1^, argon
*N*-acetyl-L-leucine amide (NALA)					
Abate el al. [7]	404.4 ± 0.2	16.55 ± 0.14		DSC	1 to 2 K min^−1^, nosp. ^b^
Ferro et al. [9]	404.0 ± 0.1	20.2 ± 0.3		DSC	1 K min^−1^, nosp. ^b^
Barone et al. [10]	401.4 ± 0.3	17.4 ± 0.3		DSC	2 K min^−1^, nosp. ^b^
This work (phase α)	406.6 ± 0.7	21.8 ± 0.7		DSC	5 K min^−1^, nitrogen
This work (phase β)	403.9 ± 1.0	19.7 ± 0.8		DSC	5 K min^−1^, nitrogen
This work (phase α)	404		510	TGA HeatFlow	5 K min^−1^, argon

^a^ Sources where melting/decomposition temperature is merely mentioned are not listed. Values determined in this work are listed together with expanded uncertainties (*k* = 2). ^b^ nosp. stands for not specified. ^c^ After the onset of decomposition.

**Table 2:** Initial crystal structures of *N*-acetyl amino acid amides studied in this work.

Compound	Phase Label	CSD Refcode ^a^	*Z*	Space Group	Ref. ^b^
*N-*acetyl glycine amide	α	JAHZEX10	4	*P*2_1_/n	Puliti et al. [12]
*N-*acetyl-L-alanine amide	crII	JAHZIB10	2	*P*2_1_	Puliti et al. [12]
*N-*acetyl-L-valine amide	crI + crII?	- ^c^			
*N-*acetyl-L-isoleucine amide	crIII	POXPEX	4	*P*2_1_	Puliti et al. [13]
*N-*acetyl-L-leucine amide	α	- ^c^			

^a^ Identifier in the Cambridge Structural Database (CSD). ^b^ Reference in which crystal structure parameters with the given CSD Refcode were determined. ^c^ Diffractograms obtained in this work are provided in Figure S1 in the Supplementary Materials (SM). They do not match the known structures JEXNAB [11] and JAHZUN10 [12] for NAVA and NALA, respectively.

**Table 3:** Solid–solid phase transitions of *N-*acetyl amides studied ^a^.

Reference	*T*_transition_/K	Δ*H*_transition_/kJ·mol^−1^	Method	Notes
*N*-acetyl-L-alanine amide, crII → crI				
This work	423.6 ± 1.0	0.5 ± 0.1	DSC	onset, 1st order
*N*-acetyl-L-valine amide, crII → crI				
This work	477.6 ± 1.0	0.3 ± 0.1	DSC	onset, 1st order
*N*-acetyl-L-isoleucine amide, crIII → crII				
This work	429.3 ± 1.0	1.5 ± 0.1	DSC	onset, 1st order
*N*-acetyl-L-isoleucine amide, crII → crI				
This work	502.0 ± 1.0	0.5 ± 0.1	DSC	onset, 1st order
*N*-acetyl-L-leucine amide, unspecified crystal → crystal phase transition				
Abate el al. [7]	382	0.3	DSC	-
Barone et al. [10]	382	0.3 ± 0.1	DSC	-

^a^ Values determined in this work are listed together with expanded uncertainties (*k* = 2).

**Table 4:** Overview of the literature on the heat capacities of *N-*acetyl amides.

Reference ^a^	*N* ^b^	(*T*_min_–*T*_max_)/K	$100ur_{\;}(C_{pm}^{o}$) ^c^	Method
*N-*acetyl glycine amide (crystal α)				
Abate et al. [7]	13	318–378	1.5	HF DSC ^d^
**This work**	**18**	**266–350**	**1.0**	**Tian-Calvet** ^e^
**This work**	**116**	**2–268**	^f^	**Relaxation** ^f^
**This work**	**32**	**216–371**	**3.0**	**PC DSC** ^g^
*N-*acetyl glycine amide (liquid)				
**This work**	**13**	**411–471**	**3.0**	**PC DSC** ^g^
*N-*acetyl-L-alanine amide (crystal crII)				
Abate et al. [7]	14	318–383	1.5	HF DSC ^d^
**This work**	**18**	**266–350**	**1.0**	**Tian-Calvet** ^e^
**This work**	**116**	**2–268**	^f^	**Relaxation** ^f^
**This work**	**40**	**241–411**	**3.0**	**PC DSC** ^g^
*N-*acetyl-L-alanine amide (liquid)				
**This work**	**7**	**441–471**	**3.0**	**PC DSC** ^g^
*N-*acetyl-L-valine amide (mixture of crystals)				
Abate et al. [7]	18	318–403	1.5	HF DSC ^d^
**This work**	**18**	**266–350**	**1.0**	**Tian-Calvet** ^e^
**This work**	**116**	**2–268**	^f^	**Relaxation** ^f^
**This work**	**52**	**216–471**	**3.0**	**PC DSC** ^g^
*N-*acetyl-L-isoleucine amide (crystal crIII)				
Abate et al. [7]	15	318–388	1.5	HF DSC ^d^
**This work**	**18**	**266–350**	**1.0**	**Tian-Calvet** ^e^
**This work**	**116**	**2–268**	^f^	**Relaxation** ^f^
**This work**	**42**	**216–421**	**3.0**	**PC DSC** ^g^
*N-*acetyl-L-isoleucine amide (crystal crII)				
**This work**	**8**	**436–471**	**3.0**	**PC DSC** ^g^
*N-*acetyl-L-leucine amide (crystal α)				
Abate et al. [7]	11	318–368	1.5	HF DSC ^d^
**This work**	**18**	**266–350**	**1.0**	**Tian-Calvet** ^e^
**This work**	**116**	**2–268**	^f^	**Relaxation** ^f^
**This work**	**36**	**216–391**	**3.0**	**PC DSC** ^g^
*N-*acetyl-L-leucine amide (liquid)				
**This work**	**13**	**411–471**	**3.0**	**PC DSC** ^g^

^a^ The data from references written in bold were fitted to Equations (1) and (2). ^b^ *N* = number of data points. ^c^ *u*_r_($C_{pm}^{o}$) stands for relative uncertainty in heat capacity, as stated by the authors. ^d^ Heat-Flux DSC, Mettler DSC 20. ^e^ SETARAM μDSCIIIa. ^f^ Quantum Design PPMS. For specification of *u*_r_($C_{pm}^{o}$) of PPMS using thermal relaxation measurement technique, see Section 3.4. ^g^ Power-Compensation DSC, PerkinElmer DSC 8500.

**Table 5:** Parameters of Equations (1) and (2) for crystal heat capacities ^a^.

*a_i_*/K^−3^	*b_i_*/K^−2^	*c_i_*/K^−1^	*d_i_*	*T_i_*/K	*T_i_*_+1_/K	*N* ^b^	*s*_r_^c^
*N-*acetyl glycine amide (α)							
8.76672 × 10^−3^	−1.33563 × 10^−1^	3.31021 × 10^−1^	9.31085	0	6	14	1.40
−6.32107 × 10^−4^	2.42384 × 10^−2^	−3.24924 × 10^−1^	8.38233	6	18	13	0.94
−8.99862 × 10^−6^	1.48251 × 10^−3^	−1.62742 × 10^−2^	6.88128	18	65	25	0.35
−3.33956 × 10^−6^	2.13702 × 10^−4^	6.34477 × 10^−2^	8.45699	65	109	18	0.28
2.72794 × 10^−7^	−2.27119 × 10^−4^	6.28574 × 10^−2^	1.13779 × 10^+1^	109	325	81	0.22
−8.12707 × 10^−6^	−5.03488 × 10^−5^	2.92427 × 10^−3^	1.71078 × 10^+1^	325	371	15	0.28
*N-*acetyl glycine amide (liquid)							
-	-	8.11012 × 10^−3^	9.14363	410	471	13	0.47
*N-*acetyl-L-alanine amide (crII)							
1.45792 × 10^−3^	3.18198 × 10^−3^	−4.53293 × 10^−1^	8.67889	0	6	14	0.84
−1.04655 × 10^−3^	2.94246 × 10^−2^	−2.57654 × 10^−1^	6.38860	6	15	10	0.87
−1.37040 × 10^−5^	1.16783 × 10^−3^	1.76779 × 10^−2^	5.69018	15	44	20	0.32
−3.15009 × 10^−7^	−2.44164 × 10^−5^	5.08370 × 10^−2^	6.85075	44	170	44	0.36
1.17010 × 10^−7^	−1.43490 × 10^−4^	2.96808 × 10^−2^	1.22384 × 10^+1^	170	411	86	0.31
*N-*acetyl-L-alanine amide (liquid)							
-	-	9.02656 × 10^−3^	5.34296	430	471	7	0.70
*N-*acetyl-L-valine amide (mixture of crystals)							
−3.94846 × 10^−3^	1.20299 × 10^−1^	−1.21923	9.27141	0	10	20	1.43
−2.91688 × 10^−5^	1.84511 × 10^−3^	2.20940 × 10^−3^	5.16053	10	32	20	0.56
−5.64281 × 10^−8^	−8.00292 × 10^−5^	4.10413 × 10^−2^	5.79158	32	226	67	0.33
1.69726 × 10^−7^	−1.12870 × 10^−4^	3.61875 × 10^−3^	1.03296 × 10^+1^	226	471	79	0.23
*N-*acetyl-L-isoleucine amide (crIII)							
3.71490 × 10^−3^	−7.16931 × 10^−2^	2.06099 × 10^−1^	7.71224	0	8	18	1.34
−4.53201 × 10^−4^	1.74646 × 10^−2^	−2.27729 × 10^−1^	6.67471	8	20	10	0.39
−1.32302 × 10^−5^	1.14933 × 10^−3^	−4.36218 × 10^−3^	5.67373	20	50	18	0.29
−1.88678 × 10^−7^	−4.13852 × 10^−5^	2.88761 × 10^−2^	6.22004	50	205	52	0.31
1.97005 × 10^−7^	−1.29121 × 10^−4^	2.44775 × 10^−3^	8.99895	205	421	78	0.12
*N-*acetyl-L-isoleucine amide (crII)							
-	-	−2.50564 × 10^−2^	5.65158	430	471	8	0.55
*N-*acetyl-L-leucine amide (α)							
−1.09439 × 10^−3^	3.60612 × 10^−2^	−3.58829 × 10^−1^	5.67932	0	11	20	1.36
8.25973 × 10^−8^	−5.37608 × 10^−5^	3.72526 × 10^−2^	4.63896	11	32	20	0.60
−2.16609 × 10^−7^	−4.85572 × 10^−5^	3.51039 × 10^−2^	5.39833	32	205	58	0.51
5.05372 × 10^−7^	−1.60977 × 10^−4^	−1.14555 × 10^−3^	8.89649	205	300	43	0.23
−7.91756 × 10^−7^	−1.69463 × 10^−5^	−1.80483 × 10^−2^	7.76814	300	391	29	0.17
−1.09439 × 10^−3^	3.60612 × 10^−2^	−3.58829 × 10^−1^	5.67932	0	11	20	1.36
*N-*acetyl-L-leucine amide (liquid)							
-	-	−1.22097 × 10^−2^	−4.79112 × 10^−1^	400	471	13	0.44

^a^ In all cases, the value of 0.13 K^−1^ was used for parameter *b* in Equation (1). ^b^ *N* stands for the number of experimental data points in the given temperature interval used for correlation. ^c^ $s_{r}=100{\left\{\sum_{i=1}^{n}{\left[(C_{pm}^{\exp}-C_{pm}^{o})/C_{pm}^{o}\right]}_{i}^{2}/\left(N-m\right)\right\}}^{1/2}$, where $C_{pm}^{\exp}$ and $C_{pm}^{o}$ is the experimental and calculated (Equations (1) and (2)) heat capacity, *N* is the number of fitted data points, and *m* is the number of independent adjustable parameters.

**Table 6:** Standard thermodynamic functions of *N-*acetyl amides at *p* = 0.1 MPa and *T* = 298.15 K ^a^.

Compound	$C_{pm}^{o}$/J·K^−1^·mol^−1^	$S_{m}^{o}$/J·K^−1^·mol^−1^	$\Delta_{0}^{T}H_{m}^{o}$/kJ·mol^−1^	$\Delta_{0}^{T}G_{m}^{o}$/kJ·mol^−1^
*N-*acetyl glycine amide	153.0	183.6	26.31	−28.42
*N-*acetyl-L-alanine amide	181.2	217.7	31.20	−33.71
*N-*acetyl-L-valine amide	227.7	265.8	38.67	−40.58
*N-*acetyl-L-isoleucine amide	254.9	287.6	42.64	−43.10
*N-*acetyl-L-leucine amide	262.6	298.2	43.39	−45.53

^a^ The combined expanded uncertainty of the calculated thermodynamic values (with a 0.95 level of confidence, *k* = 2) is *U*_c_(*X*) = 0.01 *X* at 298.15 K, where *X* represents the heat capacity or the thermodynamic property. Values are reported with one digit more than is justified by the experimental uncertainty to avoid round-off errors in calculations based on these results.

**Table 7:** Parameters of Equations (1) and (2) for the difference between amino acids and their *N-*acetyl amides in J K^−1^ mol^−1 a^.

*a_i_*/K^−3^	*b_i_*/K^−2^	*c_i_*/K^−1^	*d_i_*	*T_i_*/K	*T_i_*_+1_/K
Amino Acid → *N-*Acetyl amide					
−2.16687 × 10^−1^	3.35541	−1.79457 × 10^+1^	4.12218 × 10^1^	0	5
−5.47920 × 10^−3^	1.05101 × 10^−1^	−6.43106 × 10^−1^	8.29280	5	11
−1.57363 × 10^−4^	6.47501 × 10^−3^	2.63470 × 10^−2^	7.03428	11	24
−1.98966 × 10^−6^	3.37849 × 10^−4^	1.14914 × 10^−1^	8.12534	24	118
1.56919 × 10^−7^	−2.23235 × 10^−4^	1.25688 × 10^−1^	2.02599 × 10^1^	118	400

^a^ In all cases, the value of 0.13 K^−1^ was used for parameter *b* in Equation (1).

**Table 8:** Sample description.

Compound	CAS Number	Supplier	Purity
*N-*acetyl glycine amide (NAGA)	2620-63-5	Sigma-Aldrich	0.991 ^a^
*N-*acetyl-L-alanine amide (NAAA)	15962-47-7	Bachem	>0.99 ^b^
*N-*acetyl-L-valine amide (NAVA)	37933-88-3	Bacehm	>0.99 ^b^
*N-*acetyl-L-isoleucine amide (NAIA)	56711-06-9	Bachem	>0.99 ^b^
*N-*acetyl-L-leucine amide (NALA)	28529-34-2	Bachem	>0.99 ^b^

^a^ Mass fraction purity estimated based on elemental analysis presented in the certificate of analysis provided by the supplier. ^b^ Mole fraction purity is determined by thin layer chromatography as stated in the certificate of analysis provided by the supplier.

## Data Availability

The data presented in this study are available in the Supplementary Materials.

## Supplementary Materials

The following supporting information can be downloaded at: https://www.mdpi.com/article/10.3390/molecules28145440/s1, XRPD diffractograms of *N-*acetyl-L-valine amide and *N-*acetyl-L-leucine amide (Figure S1), DSC thermograms for the *N*-acetyl amino acid amides studied (Figures S2–S6), Experimental crystal heat capacities for the *N*-acetyl amino acid amides studied (Tables S1–S10), and Tabulated standard thermodynamic functions (heat capacity, entropy, enthalpy and Gibbs energy) for the studied *N*-acetyl amino acid amides (Tables S11–S15).
