# Supplementary material for: Heat Capacities of N-Acetyl Amides of Glycine, L-Alanine, L-Valine, L-Isoleucine, and L-Leucine
Source: Molecules. 2023 Jul 16;28(14):5440. doi: 10.3390/molecules28145440 (PMC10385853; doi:10.3390/molecules28145440)
Supplement: Supplementary file 1 [file molecules-28-05440-s001.zip › molecules-2470476-supplementary.pdf]

Supplementary Materials

Heat capacities of *N*-acetyl amides of glycine, L-alanine, L-valine, L-isoleucine, and L-leucine

Vojtěch Štejfa[1], *Václav Pokorný*[1,2], *Eliška Lieberzeitová*[1], *Jakub Havlín*[3], *Michal Fulem*[1], *Květoslav Růžička*[1,*]

[1] Department of Physical Chemistry, University of Chemistry and Technology, Prague, Technická 5, CZ−166 28 Prague 6, Czech Republic; stejfav@vscht.cz (V.Š.); fulemm@vscht.cz (M.F.); e.lieberzeit@gmail.com (E.L.)

[2] Institute of Macromolecular Chemistry, Czech Academy of Sciences, Heyrovského nám. 2, CZ−162 06 Prague 6, Czech Republic; pokorny@imc.cas.cz

[3] Central Laboratories, University of Chemistry and Technology, Prague, Technická 5, CZ−166 28 Prague 6, Czech Republic; havlinj@vscht.cz

[*] Correspondence: ruzickak@vscht.cz

Supplementary materials contain:

1) XRPD diffractograms for *N*-acetyl-L-valine amide and *N*-acetyl-L-leucine amide.
2) DSC thermograms for *N*-acetyl glycine amide, *N*-acetyl-L-alanine amide, *N*-acetyl-L-valine amide, *N*-acetyl-L-isoleucine amide, and *N*-acetyl-L-leucine amide.
3) Experimental heat capacity data of *N*-acetyl glycine amide, *N*-acetyl-L-alanine amide, *N*-acetyl-L-valine amide, *N*-acetyl-L-isoleucine amide, and *N*-acetyl-L-leucine amide measured using SETARAM µDSC IIIa, PerkinElmer DSC 8500, and Quantum Design PPMS.
4) Tabulated thermodynamic functions (heat capacity, entropy, enthalpy, Gibbs energy) of *N*-acetyl glycine amide, *N*-acetyl-L-alanine amide, *N*-acetyl-L-valine amide, *N*-acetyl-L-isoleucine amide, and *N*-acetyl-L-leucine amide.

1) XRPD diffractograms for NALA and NAVA

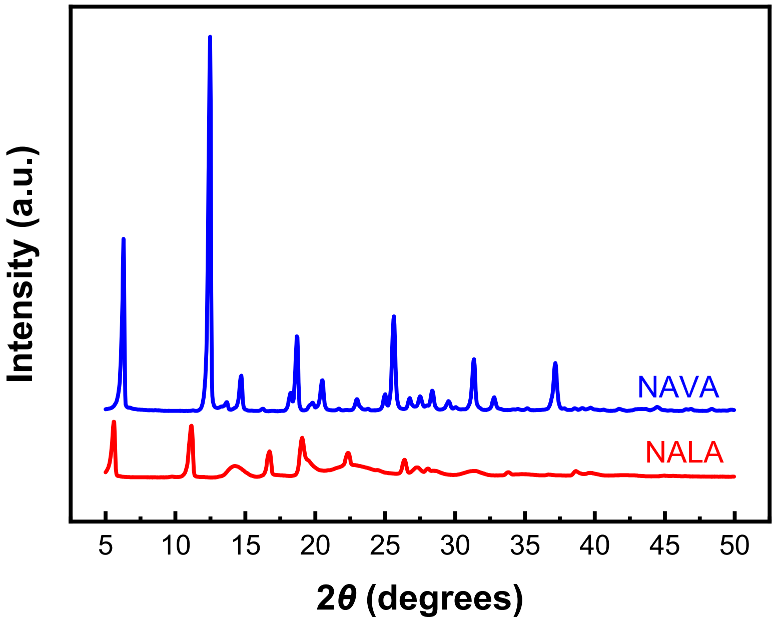

Figure S1. XRPD diffractograms for NALA and NAVA.

2) DSC Thermograms

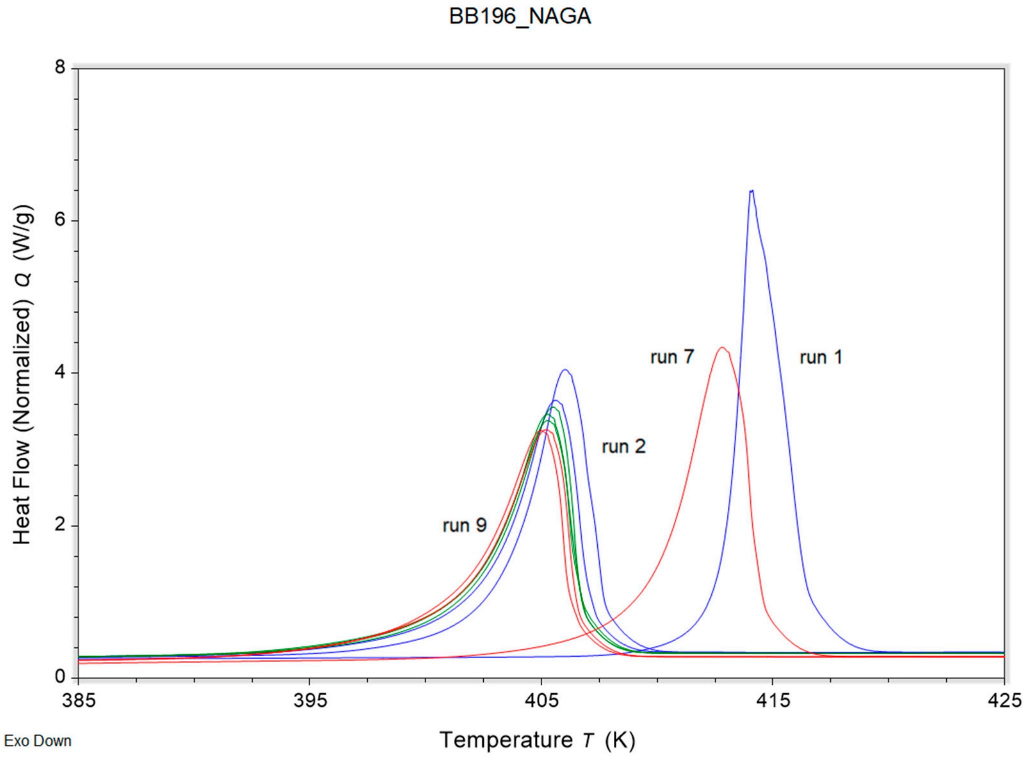

Figure S2. DSC Thermograms for NAGA. Blue: runs 1-3, green: runs 4-6, red: runs 7-9. Melting peaks in run 1 and 7 correspond to a different phase than in the other runs. The progressive shift of peaks to lower temperatures signifies slow thermal decomposition.

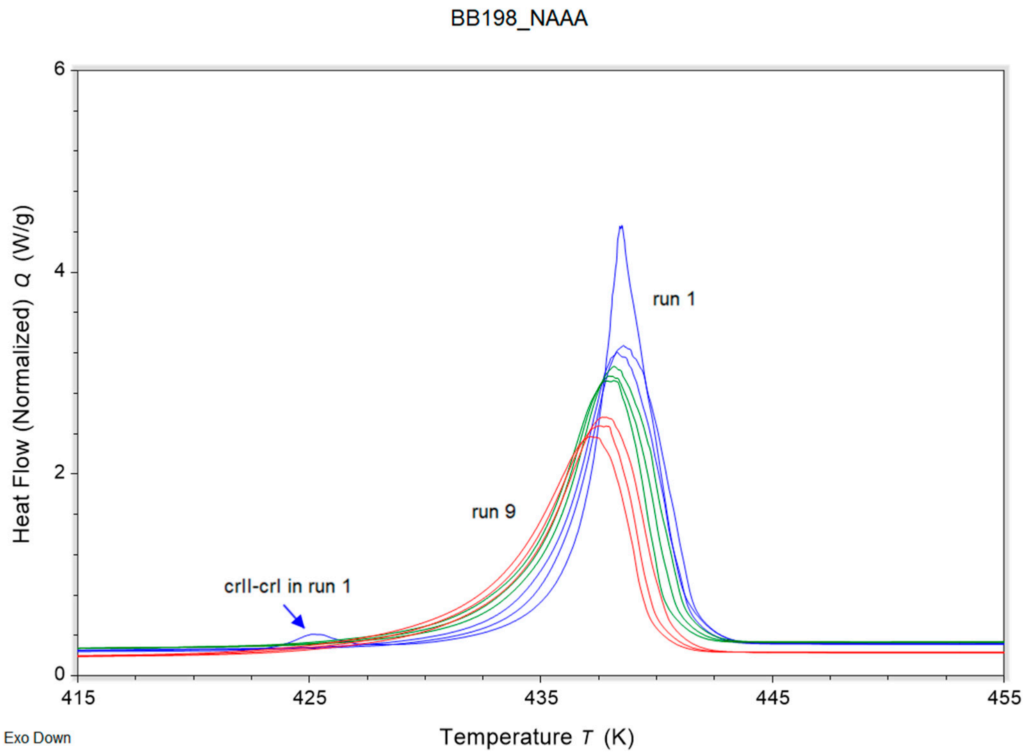

Figure S3. DSC Thermograms for NAAA. Blue: runs 1-3, green: runs 4-6, red: runs 7-9. crII-crI phase transition near 425 K was found to be partially reversible (not shown) as long as the sample was not melted for the first time.

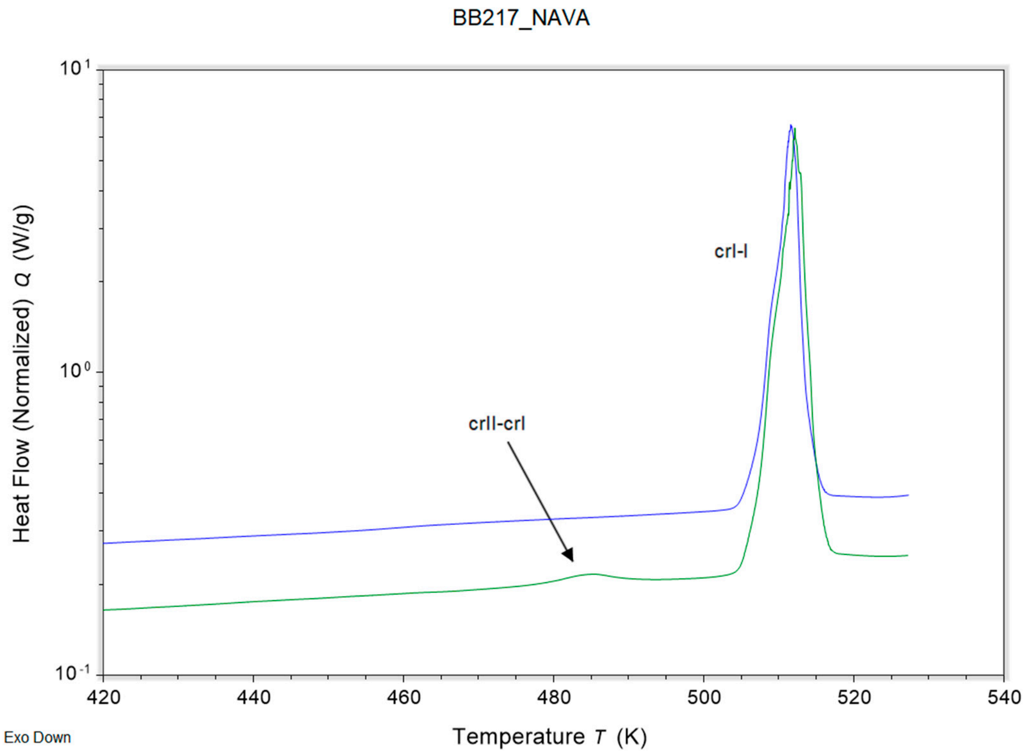

Figure S4. DSC Thermograms for NAVA. Because of abrupt thermal decomposition, both runs were performed with a freshly prepared sample.

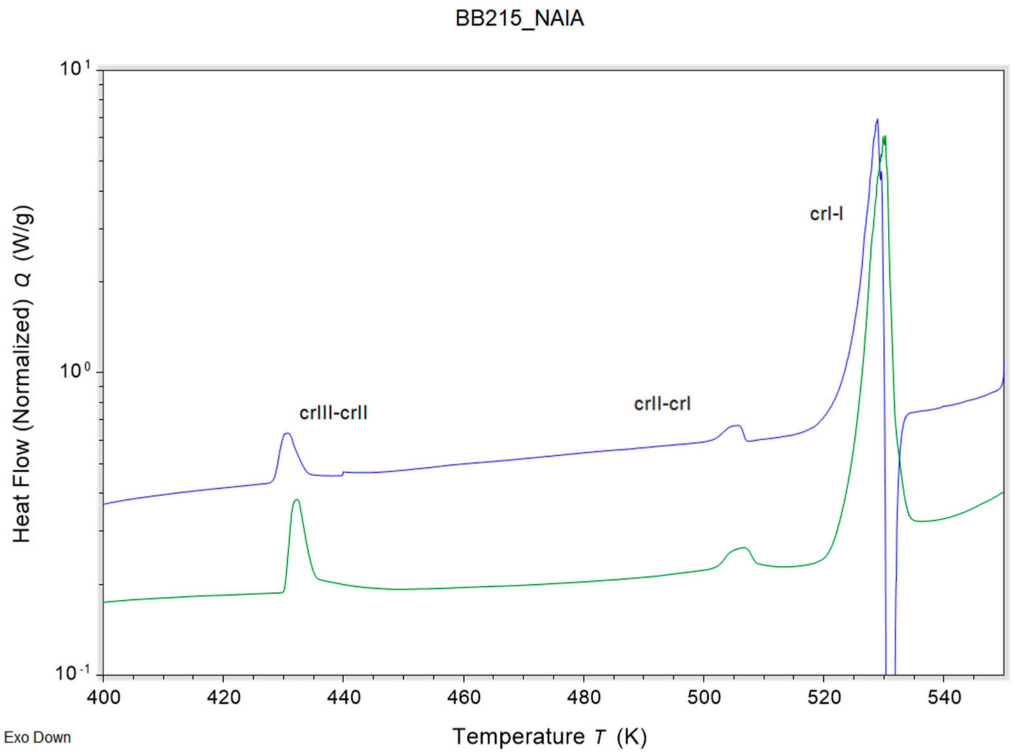

Figure S5. DSC Thermograms for NAIA. Because of abrupt thermal decomposition, both runs were performed with a freshly prepared sample.

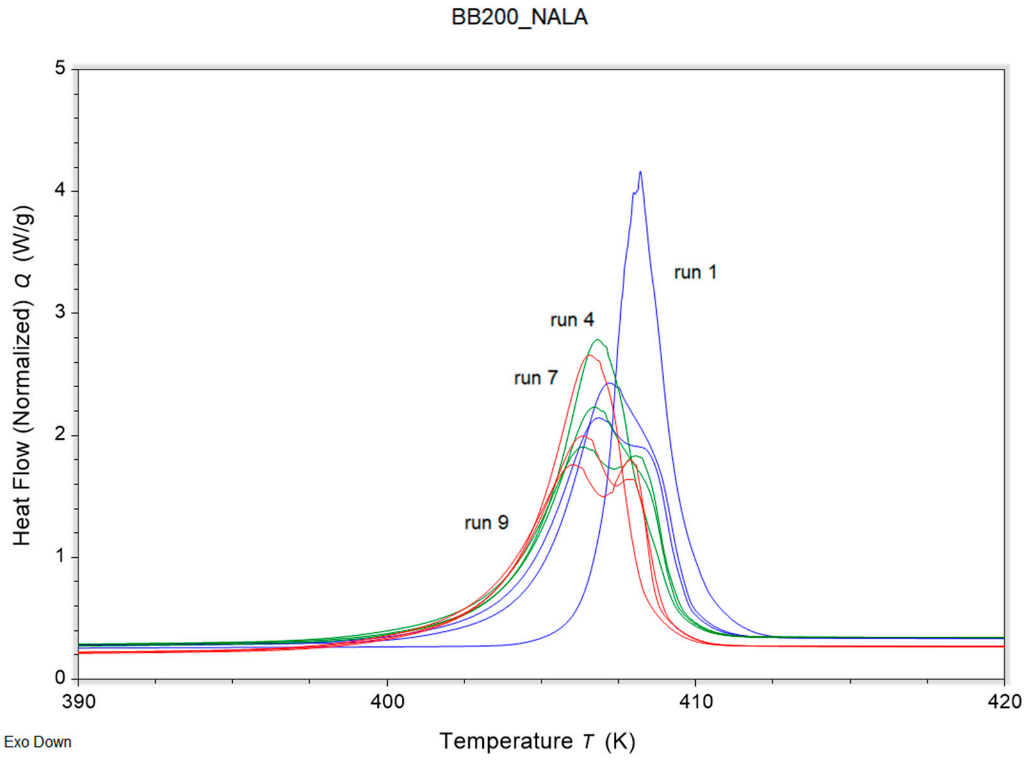

Figure S6. DSC Thermograms for NALA. Blue: runs 1-3, green: runs 4-6, red: runs 7-9. Two polymorphs can be distinguished, that occur concomitantly in runs 2, 3, 5, 6, and 8.

3) Experimental heat capacities

Table S1. Experimental molar heat capacity C^{o}_{pm} of *N*-acetyl glycine amide (in J K^{-1} mol^{-1}) at p = (100 ± 5) kPa.

SETARAM µDSC IIIa[a] (*m* = 471.70 mg)			PerkinElmer DSC 8500[b] (*m* = 10.86 mg)			
T / K	C^{o}_{pm} / J·K^{-1}·mol^{-1} [c]	$100\delta_{\text{rel}}$ [d]	T / K	C^{o}_{pm} / J·K^{-1}·mol^{-1} [c]	C^{o}_{pm} corrected [e]	$100\delta_{\text{rel}}$ [d]
Crystal α			Crystal α			
266.80	139.95	0.05	215.71	123.41	119.47	0.52
270.00	141.21	0.00	220.68	125.51	121.50	0.52
275.00	143.21	−0.07	225.67	127.61	123.53	0.52
280.00	145.21	−0.12	230.65	129.47	125.34	0.33
285.00	147.29	−0.13	235.65	131.48	127.28	0.24
290.00	149.42	−0.10	240.65	133.63	129.37	0.26
295.00	151.61	−0.03	245.65	135.78	131.44	0.27
300.00	153.76	0.00	250.67	137.93	133.52	0.26
305.00	155.84	0.00	255.68	139.98	135.51	0.20
310.00	157.94	0.01	260.70	141.68	137.15	−0.13
315.00	159.97	−0.02	265.72	143.68	139.09	−0.24
320.00	162.06	−0.01	270.75	146.39	141.71	0.13
325.00	164.23	0.05	275.76	148.74	143.99	0.26
330.00	166.40	0.12	280.76	150.65	145.84	0.09
335.00	168.56	0.14	285.77	152.03	147.08	−0.49
340.00	170.67	0.09	290.78	154.50	149.57	−0.22
345.00	172.73	−0.08	295.79	156.99	151.98	−0.01
349.89	175.01	−0.21	300.79	158.52	153.46	−0.40
			305.83	156.33	155.65	−0.34
			310.84	158.57	157.89	−0.24
			315.84	161.10	160.40	0.03
			320.85	163.00	162.29	−0.08
			325.85	165.10	164.38	−0.07
			330.85	167.45	166.72	0.09
			335.85	169.90	169.17	0.29
			340.86	171.92	171.17	0.15
			345.86	174.83	174.07	0.45
			350.87	177.36	176.59	0.39
			355.88	180.49	179.71	0.53
			360.89	183.47	182.68	0.40
			365.89	186.28	185.48	−0.02
			370.90	190.10	189.28	−0.15
			Liquid			
			410.91	283.45	282.22	−0.36
			415.92	284.78	283.55	−0.23
			420.92	287.35	286.10	0.34
			425.92	288.82	287.57	0.53
			430.92	289.58	288.32	0.48
			435.92	289.53	288.27	0.16
			440.91	289.49	288.24	−0.15
			445.90	289.54	288.28	−0.43
			450.90	290.10	288.85	−0.52
			455.90	290.92	289.66	−0.52
			460.90	293.00	291.73	−0.09
			465.91	295.69	294.40	0.55

SETARAM μDSC IIIa[a] (m = 471.70 mg)			PerkinElmer DSC 8500[b] (m = 10.86 mg)			
T / K	C^{o}_{pm} / J·K^{-1}·mol^{-1} [c]	100δ_{rel} [d]	T / K	C^{o}_{pm} / J·K^{-1}·mol^{-1} [c]	C^{o}_{pm} corrected [e]	100δ_{rel} [d]
Crystal α			Crystal α			
			470.91	295.55	294.27	0.24

[a] Standard uncertainty of temperature is $u(T)$ = 0.05 K, and the combined expanded uncertainty of the heat capacity is $U_c(C^{o}_{pm}) = 0.01\,C^{o}_{pm}$ (0.95 level of confidence).

[b] Standard uncertainty of temperature is $u(T)$ = 0.05 K, and the combined expanded uncertainty of the heat capacity is $U_c(C^{o}_{pm}) = 0.03\,C^{o}_{pm}$ (0.95 level of confidence).

[c] Values are reported with more digits than is justified by the experimental uncertainty to avoid round-off errors in calculations based on these results.

[d] $\delta_{rel} = \left(C^{o,exp}_{pm} - C^{o,calc}_{pm}\right) / C^{o,calc}_{pm}$, where $C^{o,calc}_{pm}$ is heat capacity calculated by means of Eqs. 1 and 2 with parameters from Table 5.

[e] Experimental heat capacity data from PerkinElmer DSC 8500 has been multiplied by the factor of 0.968 and 0.996 (below and above 301 K, respectively) to agree with the more accurate heat capacities obtained with SETARAM μDSC IIIa data.

Table S2. Experimental molar heat capacity C^{o}_{pm} of *N*-acetyl glycine amide obtained using the relaxation technique (Quantum Design PPMS).[a] m_{NAGA} = 8.127 mg, m_{Cu} = 19.424 mg.

T / K	C^{o}_{pm} / J·K^{-1}·mol^{-1}	100δ_{rel} [b]	T / K	C^{o}_{pm} / J·K^{-1}·mol^{-1}	100δ_{rel} [b]
Run 1, crystal α			Run 2, crystal α		
267.786	140.35	−1.98	267.863	140.25	−2.29
260.797	137.12	1.02	260.872	137.11	0.32
253.801	134.24	2.12	253.872	134.17	2.04
246.817	131.39	0.69	246.873	131.40	0.65
239.819	128.62	−0.29	239.866	128.59	−0.31
232.820	125.99	−0.31	232.862	125.88	−0.40
225.834	123.15	−1.52	225.867	123.11	−1.33
218.833	120.47	−0.32	218.874	120.33	−0.61
211.847	117.35	2.25	211.881	117.36	1.50
204.862	114.46	−0.15	204.881	114.48	−0.22
197.884	111.57	−0.28	197.883	111.67	−0.45
190.887	108.95	−0.65	190.889	109.02	−0.68
183.903	106.35	−0.25	183.893	106.38	1.01
176.905	103.64	−0.03	176.892	103.69	0.26
169.907	100.97	0.55	169.884	101.08	0.62
162.906	98.199	0.16	162.877	98.379	−0.56
155.901	95.327	0.69	155.851	95.631	−0.76
148.887	92.497	−0.04	148.837	92.732	−0.02
141.875	89.682	−0.03	141.814	90.087	0.01
134.870	87.077	−0.14	134.820	87.356	−0.17
127.851	84.375	−0.25	127.808	84.702	−0.24
120.861	81.870	−0.10	120.835	82.111	−0.11
113.855	79.152	0.13	113.830	79.486	0.14
106.865	76.487	0.62	106.828	76.712	0.28
100.797	74.471	0.10	100.800	74.485	0.15
95.800	72.492	−0.08	95.757	72.702	−0.10
90.774	70.445	−0.10	90.718	70.815	−0.37
85.695	68.335	−0.13	85.652	68.507	−0.48
80.634	65.851	0.01	80.596	66.002	0.04
75.622	63.256	0.30	75.602	63.223	0.05
70.567	60.065	0.45	70.526	60.250	0.17
65.522	56.962	0.54	65.488	57.093	−0.02
60.475	53.572	0.24	60.446	53.559	−0.07
55.380	49.641	−0.07	55.382	49.668	−0.05
50.313	45.325	−0.16	50.334	45.190	−0.48
45.264	39.995	0.00	45.266	40.003	−0.44
40.210	34.231	−0.02	40.211	34.228	−0.33
35.147	28.021	−0.06	35.150	28.029	−0.46
30.104	21.632	−0.04	30.106	21.628	−0.38
28.113	19.121	0.06	28.109	19.107	−0.41
26.076	16.546	0.05	26.077	16.551	−0.23
24.063	14.185	0.28	24.078	13.999	−0.05
22.055	11.666	0.34	22.033	11.725	0.14
20.002	9.5202	0.33	20.004	9.5293	0.21
18.011	7.4681	0.22	17.991	7.4269	0.16
15.953	5.5550	0.17	15.961	5.6320	0.14
13.939	3.9133	0.00	13.941	3.9136	0.06
11.937	2.5518	−0.04	11.941	2.5498	−0.13

T / K	C^{o}_{pm} / J·K^{-1}·mol^{-1}	$100\delta_{rel}$ [b]	T / K	C^{o}_{pm} / J·K^{-1}·mol^{-1}	$100\delta_{rel}$ [b]
Run 1, crystal α			Run 2, crystal α		
9.905	1.4559	−0.03	9.902	1.4555	−0.02
7.772	0.68329	0.05	7.808	0.68876	0.04
6.249	0.32117	0.29	6.252	0.32074	0.16
5.170	0.16725	0.15	5.172	0.16778	0.11
4.287	0.090937	0.14	4.289	0.090996	0.04
3.610	0.052251	−0.05	3.610	0.052240	−0.09
3.025	0.030296	−0.14	3.025	0.030283	−0.15
2.562	0.018455	−0.17	2.562	0.018442	−0.24
2.195	0.011449	−0.18	2.195	0.011369	−0.21
1.908	0.007311	0.05	1.908	0.007289	−0.05

[a] Standard uncertainty of temperature is $u(T)$ = 0.004 K, and the combined expanded uncertainty of heat capacity $U_c(C^{o}_{pm})$ with 0.95 level of confidence (k = 2) is $U_c(C^{o}_{pm})$ = 0.1 C^{o}_{pm} below 10 K; $U_c(C^{o}_{pm})$ = 0.03 C^{o}_{pm} in temperature range (10 to 40) K; $U_c(C^{o}_{pm})$ = 0.02 C^{o}_{pm} in temperature range (40 to 300) K. Experimental heat capacity data from Quantum Design PPMS has been multiplied by the factor of 1.003 to agree with the more accurate heat capacities obtained with SETARAM μDSC IIIa data. Values are reported with more digits than is justified by the experimental uncertainty to avoid round-off errors in calculations based on these results. Measurements are performed in vacuum (residual pressure p<10^{-4} Pa).

[b] $\delta_{rel} = \left(C^{o,exp}_{pm} - C^{o,calc}_{pm}\right) / C^{o,calc}_{pm}$, where $C^{o,calc}_{pm}$ is heat capacity calculated by means of Eqs. 1 and 2 with parameters from Table 5.

Table S3. Experimental molar heat capacity $C^{\text{o}}_{p\text{m}}$ of *N*-acetyl-L-alanine amide (in J K^{-1} mol^{-1}) at p = (100 ± 5) kPa.

SETARAM μDSC IIIa[a] (*m* = 463.31 mg)			PerkinElmer DSC 8500[b] (*m* = 12.65 mg)			
T / K	$C^{\text{o}}_{p\text{m}}$ / J·K^{-1}·mol^{-1} [c]	100δ_{rel} [d]	T / K	$C^{\text{o}}_{p\text{m}}$ / J·K^{-1}·mol^{-1} [c]	$C^{\text{o}}_{p\text{m}}$ corrected [e]	100δ_{rel} [d]
Crystal crII			Crystal crII			
266.26	165.65	0.02	215.69	140.50	140.50	−1.03
270.00	167.57	0.09	220.67	142.49	142.49	−1.22
275.00	169.95	0.07	225.65	144.84	144.85	−1.15
280.00	172.25	0.00	230.64	147.45	147.45	−0.94
285.00	174.65	−0.03	235.64	150.53	150.53	−0.42
290.00	177.12	−0.02	240.64	153.15	153.15	−0.23
295.00	179.69	0.05	245.65	155.45	155.45	−0.25
300.00	182.23	0.08	250.66	158.08	158.08	−0.08
305.00	184.73	0.08	255.67	160.61	160.61	0.02
310.00	187.19	0.06	260.69	162.60	162.60	−0.22
315.00	189.65	0.03	265.72	164.89	164.89	−0.29
320.00	192.11	−0.01	270.74	167.60	167.60	−0.10
325.00	194.67	0.00	275.76	169.73	169.73	−0.28
330.00	197.20	−0.01	280.76	172.44	172.44	−0.11
335.00	199.73	−0.03	285.76	174.93	174.93	−0.08
340.00	202.21	−0.07	290.77	178.09	178.09	0.32
345.00	204.64	−0.15	295.78	180.02	180.02	0.01
349.46	207.08	−0.09	300.79	184.01	184.01	0.84
			305.83	180.76	184.64	−0.19
			310.84	183.31	187.24	−0.14
			315.84	185.81	189.79	−0.12
			320.85	188.30	192.33	−0.12
			325.85	190.87	194.96	−0.07
			330.85	193.34	197.49	−0.08
			335.85	195.77	199.97	−0.13
			340.86	198.38	202.64	−0.08
			345.86	201.39	205.71	0.15
			350.87	203.65	208.02	0.01
			355.88	206.74	211.17	0.26
			360.89	209.88	214.38	0.53
			365.89	211.77	216.31	0.19
			370.90	214.12	218.71	0.08
			375.90	217.34	222.01	0.36
			380.90	219.49	220.59	0.15
			385.90	221.87	226.63	0.04
			390.90	223.84	228.64	−0.26
			395.90	226.58	231.44	−0.21
			400.89	229.19	234.11	−0.22
			405.90	232.43	237.41	0.03
			410.92	236.27	241.34	0.53
			liquid			
			440.93	338.08	345.33	−0.41
			445.92	340.61	347.91	0.26
			450.92	339.54	346.82	−0.13
			455.92	341.50	348.83	0.37
			460.92	342.88	350.24	0.70
			465.92	338.09	345.34	−0.78

SETARAM μDSC IIIa[a] (*m* = 463.31 mg)			PerkinElmer DSC 8500[b] (*m* = 12.65 mg)			
T / K	$C^{\text{o}}_{p\text{m}}$ / J·K^{-1}·mol^{-1} [c]	100δ_{rel} [d]	T / K	$C^{\text{o}}_{p\text{m}}$ / J·K^{-1}·mol^{-1} [c]	$C^{\text{o}}_{p\text{m}}$ corrected [e]	100δ_{rel} [d]
Crystal crII			Crystal crII			
			470.92	340.93	348.24	−0.01

[a] Standard uncertainty of temperature is $u(T)$ = 0.05 K, and the combined expanded uncertainty of the heat capacity is $U_c(C^{\text{o}}_{p\text{m}}) = 0.01\,C^{\text{o}}_{p\text{m}}$ (0.95 level of confidence).

[b] Standard uncertainty of temperature is $u(T)$ = 0.05 K, and the combined expanded uncertainty of the heat capacity is $U_c(C^{\text{o}}_{p\text{m}}) = 0.03\,C^{\text{o}}_{p\text{m}}$ (0.95 level of confidence).

[c] Values are reported with more digits than is justified by the experimental uncertainty to avoid round-off errors in calculations based on these results.

[d] $\delta_{\text{rel}} = \left(C^{\text{o,exp}}_{p\text{m}} - C^{\text{o,calc}}_{p\text{m}}\right) / C^{\text{o,calc}}_{p\text{m}}$, where $C^{\text{o,calc}}_{p\text{m}}$ is heat capacity calculated by means of Eqs. 1 and 2 with parameters from Table 5.

[e] Experimental heat capacity data from PerkinElmer DSC 8500 has been multiplied by the factor of 1.000 and 1.021 (below and above 301 K, respectively) to agree with the more accurate heat capacities obtained with SETARAM μDSC IIIa data.

Table S4. Experimental molar meat capacity C^{o}_{pm} of *N*-acetyl-L-alanine amide obtained using the relaxation technique (Quantum Design PPMS).[a] m_{NAAA} = 6.593 mg, m_{Cu} = 15.937 mg.

T / K	C^{o}_{pm} / J·K^{-1}·mol^{-1}	100δ_{rel} [b]	T / K	C^{o}_{pm} / J·K^{-1}·mol^{-1}	100δ_{rel} [b]
Run 1, crystal crII			Run 2, crystal crII		
267.757	166.44	0.06	267.883	166.46	0.03
260.768	162.87	−0.08	260.890	162.79	−0.16
253.779	159.54	−0.09	253.891	159.43	−0.19
246.786	156.41	0.02	246.892	156.23	−0.13
239.790	153.31	0.14	239.890	153.24	0.06
232.797	150.17	0.21	232.882	150.11	0.15
225.805	146.96	0.23	225.888	146.80	0.10
218.819	143.95	0.38	218.890	143.88	0.31
211.834	140.37	0.12	211.897	140.35	0.08
204.843	137.12	0.06	204.897	137.00	−0.04
197.862	133.92	0.04	197.899	133.92	0.02
190.880	130.89	0.13	190.898	130.81	0.06
183.899	127.75	0.14	183.901	127.66	0.07
176.897	124.76	0.26	176.911	124.80	0.29
169.904	121.58	0.24	169.909	121.64	0.28
162.923	118.39	0.25	162.900	118.44	0.19
155.927	114.96	0.07	155.896	115.07	−0.04
148.933	111.47	−0.06	148.881	111.73	−0.31
141.903	108.15	−0.11	141.864	108.43	−0.39
134.913	104.87	−0.16	134.873	105.08	−0.38
127.916	101.58	−0.11	127.878	101.76	−0.30
120.916	97.979	−0.16	120.869	98.200	−0.41
113.887	94.454	−0.16	113.873	94.581	−0.30
106.911	90.581	−0.18	106.866	90.773	−0.42
100.837	87.584	0.08	100.841	87.564	0.05
95.840	84.722	0.45	95.794	84.903	0.20
90.787	81.865	0.87	90.749	82.116	0.54
85.739	78.576	0.76	85.701	78.730	0.53
80.676	74.847	0.36	80.635	74.952	0.18
75.653	70.984	−0.09	75.626	71.027	−0.18
70.595	67.025	−0.36	70.573	67.022	−0.38
65.546	62.861	−0.46	65.514	62.912	−0.59
60.483	58.634	−0.49	60.486	58.616	−0.52
55.413	54.291	−0.07	55.421	54.275	−0.11
50.352	49.530	0.17	50.354	49.551	0.20
45.305	44.434	0.37	45.306	44.448	0.40
40.251	38.859	0.24	40.251	38.866	0.26
35.194	32.938	0.18	35.204	32.905	0.04
30.134	26.585	−0.05	30.134	26.589	−0.03
28.146	24.065	0.04	28.144	24.047	0.10
26.125	21.292	−0.04	26.105	21.374	−0.54
24.108	18.615	−0.27	24.087	18.689	−0.81
22.078	16.131	0.04	22.080	16.131	0.02
20.063	13.545	0.39	20.049	13.582	−0.02
18.010	11.062	0.44	18.014	11.061	0.39
15.987	8.6843	0.36	16.009	8.6737	−0.05
13.968	6.4777	−0.12	13.973	6.4820	−0.13
11.954	4.4911	−0.61	11.959	4.4896	−0.74

T / K	C^{o}_{pm} / J·K^{-1}·mol^{-1}	$100\delta_{rel}$ [b]	T / K	C^{o}_{pm} / J·K^{-1}·mol^{-1}	$100\delta_{rel}$ [b]
Run 1, crystal crII			Run 2, crystal crII		
9.917	2.7730	−0.07	9.931	2.7653	−0.74
7.816	1.4013	1.85	7.847	1.4038	0.79
6.279	0.68556	0.60	6.281	0.68669	0.66
5.193	0.35950	−0.54	5.194	0.36118	−0.15
4.304	0.19112	−0.37	4.304	0.19136	−0.25
3.620	0.10619	−0.98	3.620	0.10618	−0.99
3.029	0.058967	−0.49	3.029	0.058992	−0.45
2.564	0.034650	1.18	2.564	0.034625	1.10
2.194	0.020774	0.72	2.194	0.020818	0.94
1.906	0.012973	−1.00	1.906	0.012989	−0.89

[a] Standard uncertainty of temperature is $u(T)$ = 0.004 K, and the combined expanded uncertainty of heat capacity $U_c(C^{o}_{pm})$ with 0.95 level of confidence (k = 2) is $U_c(C^{o}_{pm})$ = 0.1 C^{o}_{pm} below 10 K; $U_c(C^{o}_{pm})$ = 0.03 C^{o}_{pm} in temperature range (10 to 40) K; $U_c(C^{o}_{pm})$ = 0.02 C^{o}_{pm} in temperature range (40 to 300) K. Experimental heat capacity data from Quantum Design PPMS has been multiplied by the factor of 1.010 to agree with the more accurate heat capacities obtained with SETARAM µDSC IIIa data. Values are reported with more digits than is justified by the experimental uncertainty to avoid round-off errors in calculations based on these results. Measurements are performed in vacuum (residual pressure p<10^{-4} Pa).

[b] $\delta_{rel} = \left(C^{o,exp}_{pm} - C^{o,calc}_{pm}\right) / C^{o,calc}_{pm}$, where $C^{o,calc}_{pm}$ is heat capacity calculated by means of Eqs. 1 and 2 with parameters from Table 5.

Table S5. Experimental molar heat capacity C^{o}_{pm} of *N*-acetyl-L-valine amide (in J K^{-1} mol^{-1}) at p = (100 ± 5) kPa.

SETARAM μDSC IIIa[a] (*m* = 448.97 mg)			PerkinElmer DSC 8500[b] (*m* = 14.47 mg)			
T / K	C^{o}_{pm} / J·K^{-1}·mol^{-1} [c]	100δ_{rel} [d]	T / K	C^{o}_{pm} / J·K^{-1}·mol^{-1} [c]	C^{o}_{pm} corrected [e]	100δ_{rel} [d]
Mixture of crystals			Mixture of crystals			
266.33	208.55	0.10	215.73	172.83	178.43	0.05
270.00	210.82	0.13	220.70	175.47	181.16	−0.05
275.00	213.75	0.08	225.69	178.63	184.42	0.14
280.00	216.65	0.02	230.67	181.15	187.03	−0.03
285.00	219.60	−0.02	235.67	183.92	189.88	−0.08
290.00	222.65	−0.02	240.66	186.97	193.03	0.02
295.00	225.77	0.00	245.67	190.23	196.39	0.22
300.00	228.99	0.07	250.68	192.61	198.86	−0.04
305.00	232.18	0.13	255.70	195.46	201.80	−0.07
310.00	235.24	0.12	260.72	198.34	204.77	−0.09
315.00	238.18	0.06	265.74	201.34	207.87	−0.05
320.00	241.32	0.09	270.76	205.00	211.65	0.30
325.00	244.30	0.04	275.77	207.08	213.79	−0.12
330.00	247.31	0.01	280.78	209.79	216.59	−0.23
335.00	250.35	0.00	285.78	212.99	219.89	−0.11
340.00	253.38	−0.02	290.79	216.71	223.74	0.25
345.00	256.51	0.00	295.80	218.54	225.63	−0.27
349.98	259.77	0.08	300.81	221.82	229.01	−0.13
			305.84	224.67	233.07	0.28
			310.86	228.18	236.71	0.52
			315.86	229.92	238.52	−0.02
			320.87	232.75	241.45	−0.08
			325.88	235.76	244.57	−0.07
			330.88	238.16	247.06	−0.30
			335.88	240.49	249.48	−0.56
			340.89	243.70	252.81	−0.46
			345.89	247.18	256.42	−0.25
			350.90	249.85	259.20	−0.36
			355.91	252.69	262.14	−0.41
			360.92	255.53	265.09	−0.45
			365.92	259.45	269.15	−0.07
			370.93	261.77	271.56	−0.31
			375.93	264.76	274.66	−0.29
			380.93	267.65	277.66	−0.29
			385.93	270.04	280.14	−0.48
			390.93	273.15	283.36	−0.40
			395.93	276.45	286.79	−0.25
			400.94	279.39	289.83	−0.23
			405.94	283.10	293.68	0.08
			410.95	285.85	296.54	0.05
			415.96	288.22	298.99	−0.11
			420.97	289.92	300.76	−0.49
			425.97	294.21	305.21	0.03
			430.97	296.38	307.46	−0.17
			435.96	299.01	310.19	−0.20
			440.96	303.19	314.52	0.29
			445.95	304.74	316.13	−0.09

SETARAM μDSC IIIa[a] (m = 448.97 mg)			PerkinElmer DSC 8500[b] (m = 14.47 mg)			
T / K	C^{o}_{pm} / J·K^{-1}·mol^{-1} [c]	100δ_{rel} [d]	T / K	C^{o}_{pm} / J·K^{-1}·mol^{-1} [c]	C^{o}_{pm} corrected [e]	100δ_{rel} [d]
Mixture of crystals			Mixture of crystals			
			450.95	307.79	319.30	0.05
			455.95	311.77	323.42	0.48
			460.95	314.92	326.69	0.66
			465.96	316.31	328.14	0.28
			470.96	317.42	329.29	−0.17

[a] Standard uncertainty of temperature is $u(T)$ = 0.05 K, and the combined expanded uncertainty of the heat capacity is $U_c(C^{o}_{pm}) = 0.01\, C^{o}_{pm}$ (0.95 level of confidence).

[b] Standard uncertainty of temperature is $u(T)$ = 0.05 K, and the combined expanded uncertainty of the heat capacity is $U_c(C^{o}_{pm}) = 0.03\, C^{o}_{pm}$ (0.95 level of confidence).

[c] Values are reported with more digits than is justified by the experimental uncertainty to avoid round-off errors in calculations based on these results.

[d] $\delta_{rel} = \left(C^{o,exp}_{pm} - C^{o,calc}_{pm}\right) / C^{o,calc}_{pm}$, where $C^{o,calc}_{pm}$ is heat capacity calculated by means of Eqs. 1 and 2 with parameters from Table 5.

[e] Experimental heat capacity data from PerkinElmer DSC 8500 has been multiplied by the factor of 1.032 and 1.037 (below and above 301 K, respectively) to agree with the more accurate heat capacities obtained with SETARAM μDSC IIIa data.

Table S6. Experimental molar heat capacity C^{o}_{pm} of *N*-acetyl-L-valine amide obtained using the relaxation technique (Quantum Design PPMS).[a] m_{NAVA} = 8.413 mg, m_{Cu} = 14.815 mg.

T / K	C^{o}_{pm} / J·K^{-1}·mol^{-1}	$100\delta_{rel}$ [b]	T / K	C^{o}_{pm} / J·K^{-1}·mol^{-1}	$100\delta_{rel}$ [b]
Run 1, mixture of crystals			Run 2, mixture of crystals		
267.600	209.24	0.07	267.705	209.05	−0.06
260.627	204.64	−0.13	260.718	204.60	−0.17
253.638	200.42	−0.15	253.720	200.32	−0.22
246.660	196.31	−0.12	246.736	196.27	−0.17
239.669	192.49	0.05	239.742	192.33	−0.06
232.677	188.54	0.14	232.747	188.44	0.07
225.696	184.33	0.09	225.753	184.20	0.00
218.710	180.48	0.22	218.760	180.24	0.07
211.730	175.90	−0.07	211.769	175.90	−0.08
204.745	171.69	−0.16	204.771	171.62	−0.21
197.758	167.50	−0.24	197.773	167.61	−0.18
190.773	163.55	−0.17	190.781	163.66	−0.10
183.783	159.53	−0.05	183.782	159.65	−0.13
176.781	155.71	0.06	176.792	155.79	0.11
169.797	151.51	0.07	169.793	151.58	0.03
162.818	147.36	0.00	162.779	147.26	0.05
155.822	142.74	−0.02	155.784	142.97	−0.20
148.823	138.02	−0.10	148.765	138.52	−0.49
141.833	133.73	−0.12	141.776	134.08	−0.41
134.816	129.36	0.01	134.775	129.74	−0.30
127.829	124.82	0.16	127.776	125.28	−0.23
120.844	120.10	0.07	120.797	120.40	−0.20
113.822	115.13	0.07	113.797	115.45	−0.22
106.840	110.18	0.08	106.807	110.33	−0.08
100.774	106.02	0.36	100.790	105.95	0.28
95.776	102.14	0.59	95.748	102.26	0.45
90.731	98.295	0.78	90.712	98.299	0.76
85.679	93.799	0.71	85.645	93.890	0.58
80.631	88.857	0.34	80.584	89.050	0.08
75.598	83.994	−0.14	75.572	84.003	−0.18
70.544	78.756	−0.38	70.512	78.934	−0.65
65.485	73.820	−0.53	65.466	73.743	−0.45
60.411	68.314	−0.66	60.413	68.340	−0.63
55.359	62.922	−0.34	55.359	62.921	−0.34
50.302	57.186	−0.03	50.309	57.174	−0.07
45.237	51.041	0.16	45.240	51.045	0.16
40.188	44.541	0.25	40.195	44.545	0.24
35.114	37.841	0.70	35.145	37.723	0.27
30.028	30.568	0.33	30.048	30.588	0.30
28.060	27.699	0.14	28.059	27.699	0.14
26.049	24.535	−0.06	26.022	24.708	−0.92
24.031	21.746	−0.38	24.015	21.725	−0.39
21.995	18.839	−0.17	21.998	18.850	−0.14
19.973	15.991	0.22	19.973	15.987	0.20
17.962	12.965	−0.02	17.933	13.078	−1.20
15.905	10.508	1.58	15.930	10.400	0.22
13.886	7.8265	0.28	13.886	7.8391	0.44
11.896	5.5535	0.05	11.894	5.5538	0.00

T / K	C^{o}_{pm} / J·K^{-1}·mol^{-1}	100δ_{rel} [b]	T / K	C^{o}_{pm} / J·K^{-1}·mol^{-1}	100δ_{rel} [b]
Run 1, mixture of crystals			Run 2, mixture of crystals		
9.862	3.5474	−1.11	9.858	3.5440	−1.11
7.709	1.9789	3.69	7.773	1.9357	−0.87
6.184	0.98148	−0.98	6.185	0.98164	−1.01
5.108	0.52673	−0.26	5.105	0.52548	−0.22
4.292	0.29159	1.30	4.292	0.29185	1.39
3.580	0.15015	−0.13	3.580	0.15023	−0.08
3.005	0.078678	−1.51	3.005	0.078687	−1.50
2.550	0.043705	−1.18	2.550	0.043696	−1.20
2.191	0.025728	−0.04	2.191	0.025733	−0.02
1.908	0.016114	1.99	1.908	0.016102	1.91

[a] Standard uncertainty of temperature is $u(T)$ = 0.004 K, and the combined expanded uncertainty of heat capacity $U_c(C^{o}_{pm})$ with 0.95 level of confidence (k = 2) is $U_c(C^{o}_{pm})$ = 0.1 C^{o}_{pm} below 10 K; $U_c(C^{o}_{pm})$ = 0.03 C^{o}_{pm} in temperature range (10 to 40) K; $U_c(C^{o}_{pm})$ = 0.02 C^{o}_{pm} in temperature range (40 to 300) K. Experimental heat capacity data from Quantum Design PPMS has been multiplied by the factor of 1.015 to agree with the more accurate heat capacities obtained with SETARAM μDSC IIIa data. Values are reported with more digits than is justified by the experimental uncertainty to avoid round-off errors in calculations based on these results. Measurements are performed in vacuum (residual pressure p<10^{-4} Pa).

[b] $\delta_{rel} = \left(C^{o,exp}_{pm} - C^{o,calc}_{pm}\right) / C^{o,calc}_{pm}$, where $C^{o,calc}_{pm}$ is heat capacity calculated by means of Eqs. 1 and 2 with parameters from Table 5.

Table S7. Experimental molar heat capacity C°_{pm} of *N*-acetyl-L-isoleucine amide (in J K^{-1} mol^{-1}) at p = (100 ± 5) kPa.

SETARAM µDSC IIIa[a] (m = 460.37 mg)			PerkinElmer DSC 8500[b] (m = 11.20 mg)			
T / K	C°_{pm} / J·K^{-1}·mol^{-1} [c]	100δ_{rel} [d]	T / K	C°_{pm} / J·K^{-1}·mol^{-1} [c]	C°_{pm} corrected [e]	100δ_{rel} [d]
crystal crIII			crystal crIII			
266.11	232.12	0.04	215.69	196.56	196.95	−0.17
270.00	234.93	0.06	220.67	200.02	200.41	−0.12
275.00	238.41	0.04	225.66	203.80	204.20	0.09
280.00	241.81	−0.03	230.65	206.75	207.16	−0.13
285.00	245.28	−0.07	235.64	210.30	210.71	−0.06
290.00	248.80	−0.09	240.64	213.66	214.08	−0.09
295.00	252.43	−0.07	245.65	216.84	217.26	−0.22
300.00	256.13	−0.03	250.66	220.80	221.23	0.01
305.00	260.01	0.08	255.67	223.89	224.32	−0.17
310.00	263.65	0.10	260.70	227.20	227.64	−0.26
315.00	266.96	−0.02	265.72	230.93	231.38	−0.16
320.00	270.94	0.12	270.74	234.90	235.36	0.02
325.00	274.33	0.04	275.76	238.27	238.73	−0.06
330.00	277.84	0.00	280.76	242.00	242.47	0.02
335.00	281.39	−0.02	285.77	245.09	245.57	−0.17
340.00	284.84	−0.08	290.78	249.78	250.27	0.28
345.00	288.43	−0.09	295.79	252.84	253.33	0.06
349.93	292.26	0.01	300.79	256.41	256.91	0.06
			305.83	259.01	260.33	−0.02
			310.83	262.77	264.11	0.04
			315.84	266.05	267.40	−0.08
			320.85	269.78	271.15	−0.03
			325.85	273.62	275.02	0.06
			330.85	276.90	278.31	−0.05
			335.85	280.71	282.14	0.02
			340.86	284.01	285.46	−0.08
			345.86	287.84	289.31	0.01
			350.87	291.46	292.95	0.02
			355.87	293.94	295.44	−0.35
			360.88	299.01	300.53	0.15
			365.89	302.57	304.11	0.15
			370.89	306.02	307.58	0.12
			375.89	309.62	311.20	0.14
			380.89	313.30	314.90	0.20
			385.89	316.33	317.94	0.05
			390.89	319.72	321.35	0.03
			395.90	322.88	324.53	−0.06
			400.87	325.97	327.63	−0.16
			405.88	329.93	331.61	0.01
			410.91	333.08	334.78	−0.06
			415.92	336.47	338.19	−0.04
			420.93	340.48	342.22	0.16
			crystal crII			
			435.92	345.41	347.18	0.72
			440.92	345.64	347.40	−0.11
			445.91	347.51	349.29	−0.45
			450.91	350.17	351.95	−0.56

SETARAM μDSC IIIa[a] (m = 460.37 mg)			PerkinElmer DSC 8500[b] (m = 11.20 mg)			
T / K	C^{o}_{pm} / J·K^{-1}·mol^{-1} [c]	100δ_{rel} [d]	T / K	C^{o}_{pm} / J·K^{-1}·mol^{-1} [c]	C^{o}_{pm} corrected [e]	100δ_{rel} [d]
crystal crIII			crystal crIII			
			455.91	354.51	356.31	−0.18
			460.91	358.50	360.32	0.11
			465.91	362.23	364.08	0.33
			470.91	364.45	366.31	0.14

[a] Standard uncertainty of temperature is $u(T)$ = 0.05 K, and the combined expanded uncertainty of the heat capacity is $U_c(C^{o}_{pm}) = 0.01\,C^{o}_{pm}$ (0.95 level of confidence).

[b] Standard uncertainty of temperature is $u(T)$ = 0.05 K, and the combined expanded uncertainty of the heat capacity is $U_c(C^{o}_{pm}) = 0.03\,C^{o}_{pm}$ (0.95 level of confidence).

[c] Values are reported with more digits than is justified by the experimental uncertainty to avoid round-off errors in calculations based on these results.

[d] $\delta_{rel} = \left(C^{o,exp}_{pm} - C^{o,calc}_{pm}\right) / C^{o,calc}_{pm}$, where $C^{o,calc}_{pm}$ is heat capacity calculated by means of Eqs. 1 and 2 with parameters from Table 5.

[e] Experimental heat capacity data from PerkinElmer DSC 8500 has been multiplied by the factor of 1.002 and 1.005 (below and above 301 K, respectively) to agree with the more accurate heat capacities obtained with SETARAM μDSC IIIa data.

Table S8. Experimental molar heat capacity C^{o}_{pm} of *N*-acetyl-L-isoleucine amide obtained using the relaxation technique (Quantum Design PPMS).[a] m_{NAIA} = 7.566 mg, m_{Cu} = 15.284 mg.

T / K	C^{o}_{pm} / J·K^{-1}·mol^{-1}	100δ_{rel} [b]	T / K	C^{o}_{pm} / J·K^{-1}·mol^{-1}	100δ_{rel} [b]
Run 1, crystal crIII			Run 2, crystal crIII		
267.635	233.12	0.01	267.764	233.01	−0.08
260.657	227.96	−0.10	260.780	227.82	−0.20
253.673	223.14	−0.08	253.789	223.04	−0.15
246.686	218.59	0.07	246.800	218.25	−0.13
239.698	213.89	0.12	239.804	213.51	−0.09
232.706	209.10	0.13	232.805	209.11	0.10
225.721	204.43	0.18	225.816	204.38	0.12
218.740	200.01	0.33	218.823	199.78	0.19
211.756	194.94	0.14	211.832	194.72	0.00
204.768	190.31	0.16	204.843	190.04	−0.01
197.784	185.43	0.02	197.845	185.39	−0.02
190.798	180.90	0.05	190.848	180.85	0.01
183.809	176.71	0.28	183.850	176.52	0.16
176.815	171.93	0.18	176.848	171.74	0.06
169.824	167.42	0.25	169.850	167.09	0.04
162.839	162.52	0.10	162.853	162.56	0.12
155.853	157.71	−0.14	155.844	157.43	0.04
148.862	152.41	−0.15	148.842	152.64	−0.31
141.873	147.21	−0.22	141.834	147.65	−0.54
134.879	142.35	−0.25	134.852	142.64	−0.47
127.877	137.28	0.00	127.845	137.85	−0.44
120.892	132.13	−0.14	120.867	132.39	−0.35
113.881	126.50	−0.16	113.856	126.86	−0.46
106.890	120.90	−0.07	106.867	121.25	−0.37
100.848	116.26	0.06	100.851	116.24	0.04
95.822	112.04	0.48	95.801	112.24	0.28
90.775	107.73	0.75	90.754	107.85	0.61
85.722	102.69	0.77	85.692	102.97	0.46
80.664	97.438	0.41	80.639	97.516	0.31
75.644	91.750	0.03	75.621	91.882	−0.14
70.588	86.104	−0.32	70.574	86.033	−0.25
65.537	80.179	−0.24	65.524	80.301	−0.41
60.483	74.096	−0.40	60.485	74.107	−0.39
55.428	67.971	−0.01	55.429	67.931	−0.07
50.372	61.301	0.09	50.374	61.320	0.12
45.331	54.310	0.21	45.339	54.271	0.12
40.275	46.749	0.09	40.287	46.707	−0.04
35.222	38.725	−0.14	35.226	38.741	−0.12
30.172	30.509	0.02	30.172	30.517	0.05
28.177	27.243	0.07	28.157	27.174	0.20
26.158	23.671	0.22	26.135	23.860	−0.74
24.136	20.561	0.06	24.139	20.554	0.01
22.120	17.302	0.21	22.110	17.367	−0.25
20.097	14.347	0.50	20.092	14.332	0.55
18.071	11.348	−0.10	18.071	11.344	−0.14
16.036	8.5733	−0.50	16.044	8.6185	−0.10
14.020	6.1060	−0.42	14.017	6.1013	−0.40

T / K	C^{o}_{pm} / J·K^{-1}·mol^{-1}	$100\delta_{rel}$ [b]	T / K	C^{o}_{pm} / J·K^{-1}·mol^{-1}	$100\delta_{rel}$ [b]
Run 1, crystal crIII			Run 2, crystal crIII		
12.003	3.9791	0.00	12.002	3.9789	−0.01
9.968	2.2726	0.19	9.974	2.2903	0.78
7.837	1.0536	1.15	7.873	1.0684	1.02
6.453	0.54307	−0.31	6.411	0.53296	−0.59
5.311	0.28602	−1.08	5.314	0.28600	−1.27
4.406	0.15873	−0.59	4.405	0.15875	−0.68
3.690	0.092354	−0.02	3.690	0.092199	−0.19
3.082	0.053939	0.69	3.082	0.053798	0.43
2.597	0.032859	2.18	2.598	0.032827	1.97
2.213	0.020240	0.92	2.213	0.020271	1.07
1.911	0.012724	−2.44	1.911	0.012722	−2.45

[a] Standard uncertainty of temperature is $u(T)$ = 0.004 K, and the combined expanded uncertainty of heat capacity $U_c(C^{o}_{pm})$ with 0.95 level of confidence (k = 2) is $U_c(C^{o}_{pm}) = 0.1\ C^{o}_{pm}$ below 10 K; $U_c(C^{o}_{pm}) = 0.03\ C^{o}_{pm}$ in temperature range (10 to 40) K; $U_c(C^{o}_{pm}) = 0.02\ C^{o}_{pm}$ in temperature range (40 to 300) K. Experimental heat capacity data from Quantum Design PPMS has been multiplied by the factor of 1.012 to agree with the more accurate heat capacities obtained with SETARAM µDSC IIIa data. Values are reported with more digits than is justified by the experimental uncertainty to avoid round-off errors in calculations based on these results. Measurements are performed in vacuum (residual pressure $p<10^{-4}$ Pa).

[b] $\delta_{rel} = \left(C^{o,exp}_{pm} - C^{o,calc}_{pm}\right) / C^{o,calc}_{pm}$, where $C^{o,calc}_{pm}$ is heat capacity calculated by means of Eqs. 1 and 2 with parameters from Table 5.

Table S9. Experimental molar heat capacity $C^{\mathrm{o}}_{p\mathrm{m}}$ of *N*-acetyl-L-leucine amide (in J K^{-1} mol^{-1}) at p = (100 ± 5) kPa.

SETARAM µDSC IIIa[a] (m = 466.60 mg)			PerkinElmer DSC 8500[b] (m = 10.01 mg)			
T / K	$C^{\mathrm{o}}_{p\mathrm{m}}$ / J·K^{-1}·mol^{-1} [c]	100δ_{rel} [d]	T / K	$C^{\mathrm{o}}_{p\mathrm{m}}$ / J·K^{-1}·mol^{-1} [c]	$C^{\mathrm{o}}_{p\mathrm{m}}$ corrected [e]	100δ_{rel} [d]
Crystal α			Crystal α			
266.22	238.34	0.07	215.69	201.61	199.70	0.04
270.00	241.35	0.10	220.67	205.23	203.28	−0.02
275.00	245.13	0.07	225.65	208.93	206.94	−0.05
280.00	248.85	0.02	230.64	212.35	210.34	−0.22
285.00	252.67	0.01	235.63	215.80	213.76	−0.39
290.00	256.58	0.06	240.63	219.54	217.46	−0.44
295.00	260.39	0.07	245.64	223.15	221.03	−0.56
300.00	264.14	0.07	250.65	227.73	225.57	−0.25
305.00	267.72	0.03	255.66	231.77	229.57	−0.19
310.00	271.24	−0.03	260.69	235.15	232.91	−0.42
315.00	274.79	−0.07	265.71	238.84	236.58	−0.51
320.00	278.40	−0.08	270.73	243.25	240.95	−0.30
325.00	282.08	−0.07	275.75	247.54	245.19	−0.14
330.00	285.86	−0.03	280.75	252.14	249.75	0.15
335.00	289.68	0.01	285.75	255.27	252.84	−0.14
340.00	293.40	0.01	290.76	260.16	257.69	0.27
345.00	297.09	−0.03	295.77	263.88	261.37	0.22
350.00	301.34	0.11	300.78	268.91	266.36	0.69
			305.84	270.65	268.11	−0.06
			310.85	274.45	271.86	−0.03
			315.85	278.68	276.06	0.17
			320.86	282.18	279.52	0.10
			325.86	285.54	282.85	−0.02
			330.86	289.59	286.86	0.10
			335.86	293.49	290.73	0.15
			340.87	296.61	293.82	−0.07
			345.87	300.27	297.44	−0.13
			350.88	303.77	300.91	−0.26
			355.89	308.40	305.50	−0.05
			360.89	312.26	309.32	−0.11
			365.90	317.01	314.03	0.08
			370.91	320.94	317.92	−0.03
			375.91	324.67	321.61	−0.24
			380.91	329.63	326.53	−0.11
			385.91	334.89	331.73	0.06
			390.91	340.02	336.81	0.14
			Liquid			
			410.93	476.47	471.99	0.17
			415.94	478.39	473.89	0.27
			420.94	480.57	476.05	0.43
			425.94	480.93	476.40	0.22
			430.94	479.41	474.90	−0.38
			435.94	480.13	475.61	−0.50
			440.94	482.45	477.91	−0.29
			445.93	482.86	478.32	−0.47
			450.92	484.64	480.08	−0.36
			455.92	486.10	481.52	−0.31

SETARAM µDSC IIIa[a] (m = 466.60 mg)			PerkinElmer DSC 8500[b] (m = 10.01 mg)			
T / K	C^{o}_{pm} / J·K^{-1}·mol^{-1} [c]	100δ$_{rel}$ [d]	T / K	C^{o}_{pm} / J·K^{-1}·mol^{-1} [c]	C^{o}_{pm} corrected [e]	100δ$_{rel}$ [d]
Crystal α			Crystal α			
			460.93	490.79	486.17	0.40
			465.93	492.22	487.58	0.45
			470.93	492.96	488.32	0.37

[a] Standard uncertainty of temperature is $u(T)$ = 0.05 K, and the combined expanded uncertainty of the heat capacity is $U_c(C^{o}_{pm}) = 0.01\, C^{o}_{pm}$ (0.95 level of confidence).

[b] Standard uncertainty of temperature is $u(T)$ = 0.05 K, and the combined expanded uncertainty of the heat capacity is $U_c(C^{o}_{pm}) = 0.03\, C^{o}_{pm}$ (0.95 level of confidence).

[c] Values are reported with more digits than is justified by the experimental uncertainty to avoid round-off errors in calculations based on these results.

[d] $\delta_{rel} = \left(C^{o,exp}_{pm} - C^{o,calc}_{pm}\right) / C^{o,calc}_{pm}$, where $C^{o,calc}_{pm}$ is heat capacity calculated by means of Eqs. 1 and 2 with parameters from Table 5.

[e] Experimental heat capacity data from PerkinElmer DSC 8500 has been multiplied by the factor of 0.991 and 0.991 (below and above 301 K, respectively) to agree with the more accurate heat capacities obtained with SETARAM µDSC IIIa data.

Table S10. Experimental molar heat capacity C^{o}_{pm} of *N*-acetyl-L-leucine amide obtained using the relaxation technique (Quantum Design PPMS).[a] m_{NALA} = 4.584 mg, m_{Cu} = 19.640 mg.

T / K	C^{o}_{pm} / J·K^{-1}·mol^{-1}	$100\delta_{rel}$ [b]	T / K	C^{o}_{pm} / J·K^{-1}·mol^{-1}	$100\delta_{rel}$ [b]
Run 1, crystal α			Run 2, crystal α		
267.778	239.28	−0.05	267.815	239.73	0.13
260.795	233.48	−0.22	260.825	233.65	−0.15
253.806	227.88	−0.30	253.832	228.08	−0.22
246.819	222.54	−0.29	246.835	222.87	−0.14
239.825	217.50	−0.14	239.826	217.87	0.03
232.812	212.53	0.03	232.838	212.75	0.13
225.834	207.24	0.15	225.830	207.48	0.03
218.845	202.36	0.36	218.837	202.67	0.20
211.864	196.71	0.00	211.839	196.77	−0.04
204.864	191.50	0.03	204.846	191.75	−0.11
197.891	186.51	−0.02	197.852	186.66	−0.12
190.895	181.64	0.17	190.861	182.09	−0.08
183.895	177.17	0.28	183.868	177.39	0.14
176.892	172.68	0.42	176.868	172.79	0.34
169.887	167.63	0.52	169.852	168.12	0.21
162.911	162.45	0.33	162.848	162.99	−0.03
155.892	156.99	0.08	155.822	157.75	−0.43
148.890	151.95	−0.02	148.816	152.75	−0.58
141.863	146.98	−0.08	141.807	147.77	−0.64
134.861	141.97	−0.01	134.805	142.90	−0.69
127.883	137.20	0.00	127.815	137.87	−0.52
120.866	132.43	0.09	120.830	132.81	−0.22
113.883	126.62	−0.11	113.818	127.21	−0.61
106.880	121.45	0.03	106.828	121.85	−0.34
100.792	117.31	0.38	100.793	117.31	0.38
95.806	113.04	0.82	95.753	113.50	0.37
90.768	108.86	1.19	90.708	109.42	0.63
85.713	103.82	1.37	85.635	104.89	0.26
80.651	98.601	0.57	80.585	99.215	−0.11
75.626	92.957	−0.23	75.568	93.424	−0.79
70.568	87.391	−0.61	70.512	87.795	−1.14
65.472	82.217	−0.59	65.471	82.295	−0.69
60.432	76.379	−0.73	60.432	76.413	−0.69
55.366	70.576	−0.24	55.367	70.641	−0.15
50.313	64.332	0.18	50.313	64.381	0.26
45.252	57.603	0.52	45.267	57.805	0.83
40.197	50.325	0.57	40.213	50.426	0.73
35.139	42.578	0.39	35.139	42.574	0.38
30.076	34.504	0.18	30.075	34.541	0.07
28.103	31.039	0.10	28.079	31.326	−0.94
26.085	27.933	−0.12	26.071	28.044	−0.59
24.056	24.659	−0.52	24.047	24.707	−0.76
22.048	21.490	−0.32	22.038	21.569	−0.76
19.997	18.451	0.07	20.000	18.470	0.15
17.973	15.364	0.12	17.976	15.380	0.20
15.978	12.456	0.25	15.971	12.436	0.32
13.917	9.6844	1.26	13.926	9.6562	0.84

T / K	C_{pm}^{o} / J·K^{-1}·mol^{-1}	100δ_{rel} [b]	T / K	C_{pm}^{o} / J·K^{-1}·mol^{-1}	100δ_{rel} [b]
Run 1, crystal α			Run 2, crystal α		
11.913	7.0725	0.84	11.911	7.0765	0.74
9.883	4.7341	−0.05	9.880	4.7340	−0.11
7.829	2.7389	−1.21	7.833	2.7464	−1.06
6.203	1.5143	−0.86	6.205	1.5156	−0.86
5.125	0.89047	−1.16	5.129	0.89121	−1.29
4.249	0.52494	0.39	4.295	0.54439	0.85
3.581	0.31525	0.60	3.581	0.31527	0.60
3.002	0.18544	1.34	3.002	0.18557	1.41
2.546	0.11209	1.83	2.546	0.11194	1.69
2.186	0.068690	0.13	2.186	0.068673	0.11
1.906	0.043562	−2.76	1.906	0.043559	−2.77

[a] Standard uncertainty of temperature is $u(T)$ = 0.004 K, and the combined expanded uncertainty of heat capacity $U_c(C_{pm}^{o})$ with 0.95 level of confidence (k = 2) is $U_c(C_{pm}^{o})$ = 0.1 C_{pm}^{o} below 10 K; $U_c(C_{pm}^{o})$ = 0.03 C_{pm}^{o} in temperature range (10 to 40) K; $U_c(C_{pm}^{o})$ = 0.02 C_{pm}^{o} in temperature range (40 to 300) K. Experimental heat capacity data from Quantum Design PPMS has been multiplied by the factor of 1.012 to agree with the more accurate heat capacities obtained with SETARAM μDSC IIIa data. Values are reported with more digits than is justified by the experimental uncertainty to avoid round-off errors in calculations based on these results. Measurements are performed in vacuum (residual pressure $p<10^{-4}$ Pa).

[b] $\delta_{rel} = \left(C_{pm}^{o,exp} - C_{pm}^{o,calc}\right) / C_{pm}^{o,calc}$, where $C_{pm}^{o,calc}$ is heat capacity calculated by means of Eqs. 1 and 2 with parameters from Table 5.

4) Tabulated thermodynamic functions

Standard thermodynamic functions were calculated using fundamental thermodynamic relationships (assuming residual entropy at 0 K for all crystalline N-acetylamino acid amides to be 0 J·K^{-1}·mol^{-1}) and heat capacities $C^{\mathrm{o}}_{p\mathrm{m}}(T)$ represented by Eqs 1 and 2 using parameters listed in Table 5:

$$S^{\mathrm{o}}_{\mathrm{m}}(T) = \int_0^T \frac{C^{\mathrm{o}}_{p\mathrm{m}}(T)}{T}\,\mathrm{d}T \qquad \text{(S1)}$$

$$\Delta_0^T H^{\mathrm{o}}_{\mathrm{m}} = \int_0^T C^{\mathrm{o}}_{p\mathrm{m}}(T)\,\mathrm{d}T \qquad \text{(S2)}$$

$$\Delta_0^T G^{\mathrm{o}}_{\mathrm{m}} = \Delta_0^T H^{\mathrm{o}}_{\mathrm{m}} - TS^{\mathrm{o}}_{\mathrm{m}}(T) \qquad \text{(S3)}$$

Table S11. Standard thermodynamic functions of *N*-acetyl glycine amide (form α)[a] at p = 0.1 MPa.[b]

T / K	$C^{\mathrm{o}}_{p\mathrm{m}}$ / J·K^{-1}·mol^{-1}	$S^{\mathrm{o}}_{\mathrm{m}}$ / J·K^{-1}·mol^{-1}	$\Delta_0^T H^{\mathrm{o}}_{\mathrm{m}}$ / kJ·mol^{-1}	$\Delta_0^T G^{\mathrm{o}}_{\mathrm{m}}$ / kJ·mol^{-1}
1[c]	1.114×10^{-3}	3.791×10^{-4}	2.830×10^{-7}	−9.611×10^{-8}
2	8.582×10^{-3}	2.907×10^{-3}	4.340×10^{-6}	−1.475×10^{-6}
3	2.932×10^{-2}	9.725×10^{-3}	2.184×10^{-5}	−7.335×10^{-6}
4	7.282×10^{-2}	2.344×10^{-2}	7.053×10^{-5}	−2.322×10^{-5}
5	1.518×10^{-1}	4.738×10^{-2}	1.793×10^{-4}	−5.761×10^{-5}
6	2.808×10^{-1}	8.560×10^{-2}	3.909×10^{-4}	−1.227×10^{-4}
7	4.719×10^{-1}	1.424×10^{-1}	7.616×10^{-4}	−2.350×10^{-4}
8	7.351×10^{-1}	2.217×10^{-1}	1.359×10^{-3}	−4.150×10^{-4}
9	1.078	3.273×10^{-1}	2.258×10^{-3}	−6.872×10^{-4}
10	1.503	4.621×10^{-1}	3.542×10^{-3}	−1.079×10^{-3}
11	2.011	6.285×10^{-1}	5.292×10^{-3}	−1.622×10^{-3}
12	2.598	8.281×10^{-1}	7.590×10^{-3}	−2.347×10^{-3}
13	3.259	1.062	1.051×10^{-2}	−3.290×10^{-3}
14	3.985	1.329	1.413×10^{-2}	−4.482×10^{-3}
15	4.771	1.631	1.850×10^{-2}	−5.960×10^{-3}
16	5.610	1.965	2.369×10^{-2}	−7.755×10^{-3}
17	6.499	2.332	2.974×10^{-2}	−9.901×10^{-3}
18	7.438	2.730	3.670×10^{-2}	−1.243×10^{-2}
19	8.428	3.158	4.463×10^{-2}	−1.537×10^{-2}
20	9.466	3.616	5.357×10^{-2}	−1.875×10^{-2}
25	15.23	6.332	1.150×10^{-1}	−4.334×10^{-2}
30	21.53	9.661	2.067×10^{-1}	−8.310×10^{-2}
35	27.91	13.46	3.304×10^{-1}	−1.407×10^{-1}
40	34.02	17.59	4.853×10^{-1}	−2.183×10^{-1}
45	39.66	21.93	6.698×10^{-1}	−3.170×10^{-1}
50	44.75	26.37	8.810×10^{-1}	−4.377×10^{-1}
55	49.27	30.86	1.116	−5.808×10^{-1}
60	53.27	35.32	1.373	−7.462×10^{-1}
65	56.83	39.73	1.648	−9.339×10^{-1}

T / K	C^{o}_{pm} / J·K^{-1}·mol^{-1}	S^{o}_{m} / J·K^{-1}·mol^{-1}	$\Delta_0^T H^{o}_{m}$ / kJ·mol^{-1}	$\Delta_0^T G^{o}_{m}$ / kJ·mol^{-1}
70	60.01	44.06	1.941	−1.143
75	62.89	48.30	2.248	−1.374
80	65.51	52.44	2.569	−1.626
85	67.90	56.48	2.903	−1.899
90	70.13	60.43	3.248	−2.191
95	72.22	64.28	3.604	−2.503
100	74.21	68.03	3.970	−2.833
110	78.04	75.29	4.731	−3.550
120	81.81	82.24	5.530	−4.338
130	85.58	88.93	6.367	−5.194
140	89.35	95.41	7.242	−6.116
150	93.13	101.7	8.154	−7.102
160	96.95	107.8	9.105	−8.150
170	100.8	113.8	10.09	−9.258
180	104.7	119.7	11.12	−10.43
190	108.6	125.5	12.19	−11.65
200	112.6	131.1	13.29	−12.94
210	116.6	136.7	14.44	−14.27
220	120.6	142.2	15.62	−15.67
230	124.7	147.7	16.85	−17.12
240	128.8	153.1	18.12	−18.62
250	132.9	158.4	19.43	−20.18
260	137.0	163.7	20.78	−21.79
270	141.2	169.0	22.17	−23.46
273.15	142.5	170.6	22.61	−23.99
280	145.4	174.2	23.60	−25.17
290	149.6	179.4	25.07	−26.94
298.15	153.0	183.6	26.31	−28.42
300	153.8	184.5	26.59	−28.76
310	157.9	189.6	28.15	−30.63
320	162.1	194.7	29.75	−32.55
330	166.2	199.7	31.39	−34.52
340	170.5	204.8	33.07	−36.54
350	175.4	209.8	34.80	−38.62
360	181.4	214.8	36.59	−40.74
370	188.8	219.9	38.44	−42.91

[a] Form α corresponds to crystal structure deposited in the Cambridge Structural Database with refcode JAHZEX10 (see Table 2 in the main article).

[b] The combined expanded uncertainty of heat capacity $U_c(C_{pm})$ as well as of all calculated thermodynamic values (with 0.95 level of confidence, k=2) is: $U_c(X)$=0.1 X below 10 K; $U_c(X)$=0.03 X in temperature range (10 to 40) K; $U_c(X)$=0.02 X in temperature range (40 to 260) K; $U_c(X)$=0.01 X in temperature range (260 to 350) K; $U_c(X)$=0.02 X in temperature range (350 to 450) K, where X represents the heat capacity or the thermodynamic property. Values are reported with one digit more than is justified by the experimental uncertainty to avoid round-off errors in calculations based on these results.

[c] Extrapolated values.

Table S12. Standard thermodynamic functions of *N*-acetyl-L-alanine amide (form crII)[a] at $p = 0.1$ MPa.[b]

T / K	C^{o}_{pm} / J·K^{-1}·mol^{-1}	S^{o}_{m} / J·K^{-1}·mol^{-1}	$\Delta_0^T H^{o}_{m}$ / kJ·mol^{-1}	$\Delta_0^T G^{o}_{m}$ / kJ·mol^{-1}
1[c]	1.711×10^{-3}	5.548×10^{-4}	4.184×10^{-7}	-1.364×10^{-7}
2	1.530×10^{-2}	4.829×10^{-3}	7.323×10^{-6}	-2.336×10^{-6}
3	5.740×10^{-2}	1.770×10^{-2}	4.046×10^{-5}	-1.265×10^{-5}
4	1.499×10^{-1}	4.539×10^{-2}	1.389×10^{-4}	-4.266×10^{-5}
5	3.182×10^{-1}	9.527×10^{-2}	3.655×10^{-4}	-1.108×10^{-4}
6	5.863×10^{-1}	1.753×10^{-1}	8.086×10^{-4}	-2.433×10^{-4}
7	9.709×10^{-1}	2.930×10^{-1}	1.577×10^{-3}	-4.740×10^{-4}
8	1.478	4.544×10^{-1}	2.791×10^{-3}	-8.439×10^{-4}
9	2.105	6.636×10^{-1}	4.574×10^{-3}	-1.399×10^{-3}
10	2.840	9.226×10^{-1}	7.038×10^{-3}	-2.188×10^{-3}
11	3.664	1.231	1.028×10^{-2}	-3.261×10^{-3}
12	4.561	1.588	1.439×10^{-2}	-4.666×10^{-3}
13	5.515	1.990	1.942×10^{-2}	-6.452×10^{-3}
14	6.518	2.436	2.544×10^{-2}	-8.661×10^{-3}
15	7.568	2.921	3.248×10^{-2}	-1.134×10^{-2}
16	8.668	3.444	4.059×10^{-2}	-1.452×10^{-2}
17	9.815	4.004	4.983×10^{-2}	-1.824×10^{-2}
18	11.00	4.598	6.023×10^{-2}	-2.254×10^{-2}
19	12.22	5.226	7.184×10^{-2}	-2.744×10^{-2}
20	13.47	5.884	8.468×10^{-2}	-3.300×10^{-2}
25	19.94	9.579	1.681×10^{-1}	-7.140×10^{-2}
30	26.43	13.79	2.841×10^{-1}	-1.297×10^{-1}
35	32.65	18.34	4.319×10^{-1}	-2.099×10^{-1}
40	38.48	23.08	6.099×10^{-1}	-3.133×10^{-1}
45	43.95	27.93	8.161×10^{-1}	-4.408×10^{-1}
50	49.10	32.83	1.049	-5.927×10^{-1}
55	53.94	37.74	1.307	-7.692×10^{-1}
60	58.49	42.63	1.588	-9.701×10^{-1}
65	62.78	47.48	1.891	−1.195
70	66.82	52.29	2.215	−1.445
75	70.63	57.03	2.559	−1.718
80	74.24	61.70	2.921	−2.015
85	77.67	66.31	3.301	−2.335
90	80.93	70.84	3.698	−2.678
95	84.04	75.30	4.110	−3.043
100	87.03	79.69	4.538	−3.431
110	92.66	88.25	5.437	−4.271
120	97.92	96.54	6.390	−5.195
130	102.9	104.6	7.394	−6.201
140	107.7	112.4	8.447	−7.286
150	112.3	120.0	9.547	−8.447
160	116.9	127.4	10.69	−9.684
170	121.3	134.6	11.88	−10.99
180	125.8	141.6	13.12	−12.38
190	130.3	148.6	14.40	−13.83
200	134.8	155.4	15.73	−15.35
210	139.4	162.1	17.10	−16.93
220	143.9	168.6	18.51	−18.59
230	148.6	175.1	19.98	−20.31
240	153.2	181.6	21.49	−22.09

T / K	C^{o}_{pm} / J·K^{-1}·mol^{-1}	S^{o}_{m} / J·K^{-1}·mol^{-1}	$\Delta_0^T H^{o}_{m}$ / kJ·mol^{-1}	$\Delta_0^T G^{o}_{m}$ / kJ·mol^{-1}
250	157.9	187.9	23.04	−23.94
260	162.6	194.2	24.64	−25.85
270	167.4	200.4	26.29	−27.82
273.15	168.9	202.4	26.82	−28.45
280	172.3	206.6	27.99	−29.86
290	177.2	212.7	29.74	−31.95
298.15	181.2	217.7	31.20	−33.71
300	182.1	218.8	31.53	−34.11
310	187.1	224.9	33.38	−36.33
320	192.1	230.9	35.28	−38.61
330	197.2	236.9	37.22	−40.95
340	202.4	242.8	39.22	−43.34
350	207.6	248.8	41.27	−45.80
360	212.8	254.7	43.37	−48.32
370	218.1	260.6	45.53	−50.90
380	223.4	266.5	47.73	−53.53
390	228.8	272.4	49.99	−56.23
400	234.2	278.2	52.31	−58.98
410	239.6	284.1	54.68	−61.79

[a] Form crII corresponds to crystal structure deposited in the Cambridge Structural Database with refcode JAHZIB10 (see Table 3 in the main article).

[b] The combined expanded uncertainty of heat capacity $U_c(C_{pm})$ as well as of all calculated thermodynamic values (with 0.95 level of confidence, k=2) is: $U_c(X)$=0.1 X below 10 K; $U_c(X)$=0.03 X in temperature range (10 to 40) K; $U_c(X)$=0.02 X in temperature range (40 to 260) K; $U_c(X)$=0.01 X in temperature range (260 to 350) K; $U_c(X)$=0.02 X in temperature range (350 to 450) K, where X represents the heat capacity or the thermodynamic property. Values are reported with one digit more than is justified by the experimental uncertainty to avoid round-off errors in calculations based on these results.

[c] Extrapolated values.

Table S13. Standard thermodynamic functions of *N*-acetyl-L-valine amide in the crystal state[a] at p = 0.1 MPa.[b]

T / K	C_{pm}^{o} / J·K^{-1}·mol^{-1}	S_{m}^{o} / J·K^{-1}·mol^{-1}	$\Delta_0^T H_m^o$ / kJ·mol^{-1}	$\Delta_0^T G_m^o$ / kJ·mol^{-1}
1[c]	1.750×10^{-3}	5.395×10^{-4}	4.110×10^{-7}	−1.285×10^{-7}
2	1.864×10^{-2}	5.433×10^{-3}	8.377×10^{-6}	−2.489×10^{-6}
3	7.940×10^{-2}	2.239×10^{-2}	5.218×10^{-5}	−1.497×10^{-5}
4	2.240×10^{-1}	6.253×10^{-2}	1.951×10^{-4}	−5.497×10^{-5}
5	4.907×10^{-1}	1.386×10^{-1}	5.411×10^{-4}	−1.520×10^{-4}
6	9.002×10^{-1}	2.621×10^{-1}	1.224×10^{-3}	−3.480×10^{-4}
7	1.448	4.403×10^{-1}	2.388×10^{-3}	−6.945×10^{-4}
8	2.112	6.759×10^{-1}	4.160×10^{-3}	−1.248×10^{-3}
9	2.869	9.676×10^{-1}	6.643×10^{-3}	−2.065×10^{-3}
10	3.709	1.313	9.924×10^{-3}	−3.201×10^{-3}
11	4.641	1.709	1.409×10^{-2}	−4.707×10^{-3}
12	5.664	2.156	1.924×10^{-2}	−6.636×10^{-3}
13	6.767	2.652	2.545×10^{-2}	−9.036×10^{-3}
14	7.942	3.197	3.279×10^{-2}	−1.196×10^{-2}
15	9.179	3.786	4.135×10^{-2}	−1.544×10^{-2}
16	10.47	4.420	5.117×10^{-2}	−1.954×10^{-2}
17	11.80	5.094	6.230×10^{-2}	−2.430×10^{-2}
18	13.17	5.807	7.479×10^{-2}	−2.974×10^{-2}
19	14.57	6.557	8.866×10^{-2}	−3.592×10^{-2}
20	15.99	7.341	1.039×10^{-1}	−4.287×10^{-2}
25	23.24	11.69	2.020×10^{-1}	−9.017×10^{-2}
30	30.43	16.56	3.362×10^{-1}	−1.606×10^{-1}
35	37.42	21.78	5.059×10^{-1}	−2.564×10^{-1}
40	44.18	27.22	7.101×10^{-1}	−3.788×10^{-1}
45	50.66	32.80	9.473×10^{-1}	−5.288×10^{-1}
50	56.84	38.46	1.216	−7.069×10^{-1}
55	62.73	44.16	1.515	−9.135×10^{-1}
60	68.32	49.86	1.843	−1.149
65	73.65	55.54	2.198	−1.412
70	78.73	61.18	2.579	−1.704
75	83.58	66.78	2.985	−2.024
80	88.22	72.33	3.415	−2.372
85	92.67	77.81	3.867	−2.747
90	96.94	83.23	4.341	−3.150
95	101.1	88.58	4.836	−3.579
100	105.0	93.86	5.351	−4.035
110	112.6	104.2	6.440	−5.026
120	119.8	114.3	7.602	−6.119
130	126.6	124.2	8.834	−7.312
140	133.1	133.8	10.13	−8.602
150	139.4	143.2	11.50	−9.988
160	145.6	152.4	12.92	−11.47
170	151.6	161.4	14.41	−13.04
180	157.5	170.3	15.95	−14.69
190	163.4	178.9	17.56	−16.44
200	169.2	187.5	19.22	−18.27
210	175.0	195.9	20.94	−20.19
220	180.8	204.1	22.72	−22.19
230	186.7	212.3	24.56	−24.27
240	192.6	220.4	26.45	−26.43

T / K	C^{o}_{pm} / J·K^{-1}·mol^{-1}	S^{o}_{m} / J·K^{-1}·mol^{-1}	$\Delta_0^T H^{o}_{m}$ / kJ·mol^{-1}	$\Delta_0^T G^{o}_{m}$ / kJ·mol^{-1}
250	198.5	228.4	28.41	−28.68
260	204.5	236.3	30.43	−31.00
270	210.6	244.1	32.50	−33.40
273.15	212.5	246.5	33.17	−34.18
280	216.6	251.9	34.64	−35.88
290	222.7	259.6	36.83	−38.44
298.15	227.7	265.8	38.67	−40.58
300	228.8	267.2	39.09	−41.07
310	235.0	274.8	41.41	−43.78
320	241.1	282.4	43.79	−46.57
330	247.3	289.9	46.23	−49.43
340	253.4	297.4	48.74	−52.37
350	259.6	304.8	51.30	−55.38
360	265.7	312.2	53.93	−58.46
370	271.8	319.6	56.61	−61.62
380	277.9	326.9	59.36	−64.85
390	284.0	334.2	62.17	−68.16
400	289.9	341.5	65.04	−71.54
410	295.8	348.7	67.97	−74.99
420	301.7	355.9	70.96	−78.51
430	307.4	363.0	74.00	−82.11
440	313.1	370.2	77.11	−85.77
450	318.6	377.3	80.27	−89.51
460	324.1	384.3	83.48	−93.32
470	329.3	391.4	86.75	−97.20

[a] Crystal structure of the sample was not resolved. The XRPD diffractogram (Figure S1) suggests that the sample might be a mixture of the form whose crystal structure is deposited in the Cambridge Structural Database with refcode JEXNAB and another (unknown) polymorph (see Table 3 in the main article).

[b] The combined expanded uncertainty of heat capacity $U_c(C_{pm})$ as well as of all calculated thermodynamic values (with 0.95 level of confidence, k=2) is: $U_c(X)$=0.1 X below 10 K; $U_c(X)$=0.03 X in temperature range (10 to 40) K; $U_c(X)$=0.02 X in temperature range (40 to 260) K; $U_c(X)$=0.01 X in temperature range (260 to 350) K; $U_c(X)$=0.02 X in temperature range (350 to 420) K, where X represents the heat capacity or the thermodynamic property. Values are reported with one digit more than is justified by the experimental uncertainty to avoid round-off errors in calculations based on these results.

[c] Extrapolated values.

Table S14. Standard thermodynamic functions of *N*-acetyl-L-isoleucine amide (form crIII)[a] at $p = 0.1$ MPa.[b]

T / K	C^{o}_{pm} / J·K^{-1}·mol^{-1}	S^{o}_{m} / J·K^{-1}·mol^{-1}	$\Delta_0^T H^{o}_{m}$ / kJ·mol^{-1}	$\Delta_0^T G^{o}_{m}$ / kJ·mol^{-1}
1[c]	1.968×10^{-3}	6.703×10^{-4}	5.004×10^{-7}	-1.700×10^{-7}
2	1.490×10^{-2}	5.097×10^{-3}	7.596×10^{-6}	-2.597×10^{-6}
3	4.941×10^{-2}	1.675×10^{-2}	3.747×10^{-5}	-1.277×10^{-5}
4	1.183×10^{-1}	3.940×10^{-2}	1.179×10^{-4}	-3.975×10^{-5}
5	2.383×10^{-1}	7.757×10^{-2}	2.911×10^{-4}	-9.672×10^{-5}
6	4.298×10^{-1}	1.367×10^{-1}	6.183×10^{-4}	-2.019×10^{-4}
7	7.156×10^{-1}	2.231×10^{-1}	1.182×10^{-3}	-3.792×10^{-4}
8	1.115	3.433×10^{-1}	2.088×10^{-3}	-6.593×10^{-4}
9	1.639	5.037×10^{-1}	3.454×10^{-3}	-1.079×10^{-3}
10	2.291	7.089×10^{-1}	5.408×10^{-3}	-1.682×10^{-3}
11	3.073	9.629×10^{-1}	8.079×10^{-3}	-2.513×10^{-3}
12	3.977	1.268	1.159×10^{-2}	-3.625×10^{-3}
13	4.993	1.626	1.607×10^{-2}	-5.067×10^{-3}
14	6.107	2.036	2.161×10^{-2}	-6.894×10^{-3}
15	7.304	2.498	2.831×10^{-2}	-9.157×10^{-3}
16	8.570	3.009	3.624×10^{-2}	-1.191×10^{-2}
17	9.892	3.568	4.547×10^{-2}	-1.519×10^{-2}
18	11.26	4.172	5.604×10^{-2}	-1.906×10^{-2}
19	12.67	4.819	6.801×10^{-2}	-2.355×10^{-2}
20	14.13	5.506	8.140×10^{-2}	-2.871×10^{-2}
25	21.95	9.484	1.713×10^{-1}	-6.582×10^{-2}
30	30.22	14.21	3.016×10^{-1}	-1.248×10^{-1}
35	38.42	19.49	4.733×10^{-1}	-2.089×10^{-1}
40	46.29	25.14	6.853×10^{-1}	-3.203×10^{-1}
45	53.72	31.03	9.355×10^{-1}	-4.607×10^{-1}
50	60.74	37.05	1.222	-6.308×10^{-1}
55	67.42	43.16	1.542	-8.313×10^{-1}
60	73.80	49.30	1.896	−1.062
65	79.87	55.45	2.280	−1.324
70	85.66	61.58	2.694	−1.617
75	91.18	67.68	3.136	−1.940
80	96.46	73.73	3.605	−2.294
85	101.5	79.73	4.100	−2.677
90	106.3	85.67	4.620	−3.091
95	111.0	91.55	5.163	−3.534
100	115.5	97.36	5.729	−4.006
110	123.9	108.8	6.927	−5.037
120	131.9	119.9	8.206	−6.181
130	139.5	130.8	9.563	−7.434
140	146.7	141.4	10.99	−8.795
150	153.7	151.7	12.50	−10.26
160	160.5	161.8	14.07	−11.83
170	167.1	171.8	15.71	−13.50
180	173.7	181.5	17.41	−15.26
190	180.3	191.1	19.18	−17.13
200	186.9	200.5	21.02	−19.08
210	193.5	209.8	22.92	−21.14
220	200.2	218.9	24.89	−23.28
230	207.0	228.0	26.92	−25.51
240	213.8	236.9	29.02	−27.84

T / K	C^{o}_{pm} / J·K^{-1}·mol^{-1}	S^{o}_{m} / J·K^{-1}·mol^{-1}	$\Delta_0^T H^{o}_{m}$ / kJ·mol^{-1}	$\Delta_0^T G^{o}_{m}$ / kJ·mol^{-1}
250	220.8	245.8	31.20	−30.25
260	227.7	254.6	33.44	−32.75
270	234.8	263.3	35.75	−35.34
273.15	237.0	266.1	36.50	−36.18
280	241.9	272.0	38.14	−38.02
290	249.0	280.6	40.59	−40.78
298.15	254.9	287.6	42.64	−43.10
300	256.2	289.2	43.12	−43.63
310	263.4	297.7	45.71	−46.57
320	270.6	306.2	48.38	−49.59
330	277.9	314.6	51.13	−52.69
340	285.1	323.0	53.94	−55.88
350	292.3	331.4	56.83	−59.15
360	299.5	339.7	59.79	−62.50
370	306.6	348.0	62.82	−65.94
380	313.7	356.3	65.92	−69.46
390	320.7	364.5	69.09	−73.07
400	327.6	372.7	72.33	−76.75
410	334.4	380.9	75.64	−80.52
420	341.0	389.0	79.02	−84.37

[a] Form crIII corresponds to crystal structure deposited in the Cambridge Structural Database with refcode POXPEX (see Table 3 in the main article).

[b] The combined expanded uncertainty of heat capacity $U_c(C_{pm})$ as well as of all calculated thermodynamic values (with 0.95 level of confidence, k=2) is: $U_c(X)$=0.1 X below 10 K; $U_c(X)$=0.03 X in temperature range (10 to 40) K; $U_c(X)$=0.02 X in temperature range (40 to 260) K; $U_c(X)$=0.01 X in temperature range (260 to 350) K; $U_c(X)$=0.02 X in temperature range (350 to 420) K, where X represents the heat capacity or the thermodynamic property. Values are reported with one digit more than is justified by the experimental uncertainty to avoid round-off errors in calculations based on these results.

[c] Extrapolated values.

Table S15. Standard thermodynamic functions of *N*-acetyl-L-leucine amide in the crystal state[a] at p = 0.1 MPa.[b]

T / K	C^{o}_{pm} / J·K^{-1}·mol^{-1}	S^{o}_{m}/ J·K^{-1}·mol^{-1}	$\Delta_0^T H^{o}_{m}$/ kJ·mol^{-1}	$\Delta_0^T G^{o}_{m}$/ kJ·mol^{-1}
1[c]	6.059×10^{-3}	1.973×10^{-3}	1.487×10^{-6}	-4.860×10^{-7}
2	5.203×10^{-2}	1.677×10^{-2}	2.535×10^{-5}	-8.198×10^{-6}
3	1.826×10^{-1}	5.900×10^{-2}	1.338×10^{-4}	-4.318×10^{-5}
4	4.370×10^{-1}	1.431×10^{-1}	4.320×10^{-4}	-1.402×10^{-4}
5	8.397×10^{-1}	2.810×10^{-1}	1.058×10^{-3}	-3.473×10^{-4}
6	1.396	4.810×10^{-1}	2.163×10^{-3}	-7.228×10^{-4}
7	2.097	7.471×10^{-1}	3.898×10^{-3}	-1.331×10^{-3}
8	2.921	1.080	6.398×10^{-3}	-2.239×10^{-3}
9	3.849	1.476	9.775×10^{-3}	-3.512×10^{-3}
10	4.862	1.934	1.412×10^{-2}	-5.212×10^{-3}
11	5.954	2.448	1.953×10^{-2}	-7.398×10^{-3}
12	7.125	3.015	2.606×10^{-2}	-1.013×10^{-2}
13	8.368	3.634	3.380×10^{-2}	-1.345×10^{-2}
14	9.675	4.302	4.282×10^{-2}	-1.741×10^{-2}
15	11.04	5.016	5.317×10^{-2}	-2.207×10^{-2}
16	12.45	5.773	6.491×10^{-2}	-2.746×10^{-2}
17	13.90	6.571	7.808×10^{-2}	-3.362×10^{-2}
18	15.39	7.407	9.272×10^{-2}	-4.061×10^{-2}
19	16.90	8.279	1.089×10^{-1}	-4.845×10^{-2}
20	18.44	9.185	1.265×10^{-1}	-5.718×10^{-2}
25	26.36	14.15	2.384×10^{-1}	-1.152×10^{-1}
30	34.36	19.66	3.903×10^{-1}	-1.996×10^{-1}
35	42.20	25.55	5.818×10^{-1}	-3.125×10^{-1}
40	49.75	31.68	8.118×10^{-1}	-4.555×10^{-1}
45	56.95	37.96	1.079	-6.295×10^{-1}
50	63.80	44.32	1.381	-8.352×10^{-1}
55	70.28	50.71	1.716	−1.073
60	76.43	57.09	2.083	−1.342
65	82.25	63.44	2.480	−1.644
70	87.79	69.74	2.905	−1.977
75	93.05	75.97	3.357	−2.341
80	98.08	82.14	3.835	−2.736
85	102.9	88.23	4.338	−3.162
90	107.5	94.24	4.864	−3.618
95	111.9	100.2	5.412	−4.104
100	116.2	106.0	5.983	−4.620
110	124.4	117.5	7.186	−5.738
120	132.1	128.6	8.468	−6.969
130	139.5	139.5	9.826	−8.310
140	146.6	150.1	11.26	−9.758
150	153.6	160.5	12.76	−11.31
160	160.5	170.6	14.33	−12.97
170	167.4	180.5	15.97	−14.72
180	174.2	190.3	17.68	−16.58
190	181.2	199.9	19.45	−18.53
200	188.2	209.4	21.30	−20.57
210	195.4	218.7	23.22	−22.71
220	202.8	228.0	25.21	−24.95
230	210.3	237.2	27.27	−27.27
240	217.9	246.3	29.42	−29.69

T / K	C^{o}_{pm} / J·K^{-1}·mol^{-1}	S^{o}_{m}/ J·K^{-1}·mol^{-1}	$\Delta_0^T H^{o}_{m}$/ kJ·mol^{-1}	$\Delta_0^T G^{o}_{m}$/ kJ·mol^{-1}
250	225.6	255.3	31.63	−32.20
260	233.4	264.3	33.93	−34.80
270	241.1	273.3	36.30	−37.49
273.15	243.5	276.1	37.06	−38.35
280	248.8	282.2	38.75	−40.26
290	256.4	291.1	41.28	−43.13
298.15	262.6	298.2	43.39	−45.53
300	264.0	299.9	43.88	−46.08
310	271.3	308.7	46.56	−49.13
320	278.6	317.4	49.31	−52.26
330	286.0	326.1	52.13	−55.47
340	293.4	334.7	55.02	−58.78
350	301.0	343.3	58.00	−62.17
360	308.9	351.9	61.05	−65.64
370	317.3	360.5	64.18	−69.21
380	326.1	369.1	67.39	−72.85
390	335.5	377.7	70.70	−76.59

[a] Crystal structure of the sample was not resolved. The XRPD diffractogram (Figure S1) shows that the sample does not correspond to crystal structure deposited in the Cambridge Structural Database with refcode JAHZUN10. It might correspond to a mixture of polymorphs or to incompletely or imperfectly crystallized sample (see Table 3 in the main article).

[b] The combined expanded uncertainty of heat capacity $U_c(C_{pm})$ as well as of all calculated thermodynamic values (with 0.95 level of confidence, k=2) is: $U_c(X)$=0.1 X below 10 K; $U_c(X)$=0.03 X in temperature range (10 to 40) K; $U_c(X)$=0.02 X in temperature range (40 to 260) K; $U_c(X)$=0.01 X in temperature range (260 to 350) K; $U_c(X)$=0.02 X in temperature range (350 to 430) K, where X represents the heat capacity or the thermodynamic property. Values are reported with one digit more than is justified by the experimental uncertainty to avoid round-off errors in calculations based on these results.

[c] Extrapolated values.
